# The recent progress of myeloid‐derived suppressor cell and its targeted therapies in cancers

**DOI:** 10.1002/mco2.323

**Published:** 2023-08-02

**Authors:** Ruiyang Ren, Chenyi Xiong, Runyu Ma, Yixuan Wang, Tianyang Yue, Jiayun Yu, Bin Shao

**Affiliations:** ^1^ State Key Laboratory of Oral Diseases & National Clinical Research Center for Oral Diseases Department of Orthodontics West China Hospital of Stomatology Sichuan University Chengdu Sichuan China; ^2^ State Key Laboratory of Oral Diseases & National Clinical Research Center for Oral Diseases West China Hospital of Stomatology Sichuan University Chengdu Sichuan China; ^3^ Department of Radiotherapy Cancer Center and State Key Laboratory of Biotherapy West China Hospital Sichuan University Chengdu China; ^4^ State Key Laboratory of Biotherapy West China Hospital Sichuan University Chengdu Sichuan China

**Keywords:** inflammation, MDSC‐targeted drugs, myeloid‐derived suppressor cells, targeted therapy, tumor microenvironment

## Abstract

Myeloid‐derived suppressor cells (MDSCs) are an immature group of myeloid‐derived cells generated from myeloid cell precursors in the bone marrow. MDSCs appear almost exclusively in pathological conditions, such as tumor progression and various inflammatory diseases. The leading function of MDSCs is their immunosuppressive ability, which plays a crucial role in tumor progression and metastasis through their immunosuppressive effects. Since MDSCs have specific molecular features, and only a tiny amount exists in physiological conditions, MDSC‐targeted therapy has become a promising research direction for tumor treatment with minimal side effects. In this review, we briefly introduce the classification, generation and maturation process, and features of MDSCs, and detail their functions under various circumstances. The present review specifically demonstrates the environmental specificity of MDSCs, highlighting the differences between MDSCs from cancer and healthy individuals, as well as tumor‐infiltrating MDSCs and circulating MDSCs. Then, we further describe recent advances in MDSC‐targeted therapies. The existing and potential targeted drugs are divided into three categories, monoclonal antibodies, small‐molecular inhibitors, and peptides. Their targeting mechanisms and characteristics have been summarized respectively. We believe that a comprehensive in‐depth understanding of MDSC‐targeted therapy could provide more possibilities for the treatment of cancer.

## Graphical abstract

Myeloid‐derived suppressor cells play a crucial role in tumor progression and metastasis through their immunosuppressive effects. In different environments and conditions, these cells vary in mechanisms of immunosuppressive functions. Since only a small amount exists in physiological conditions, myeloid‐derived suppressor cell‐targeted therapy has become a promising research orientation for tumor treatment with minimal side effects. In recent years, various kinds of targeted drugs against myeloid‐derived suppressor cells have been developed, as a novel way of cancer treatment.

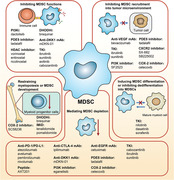

## 1 INTRODUCTION

The immune system is closely associated with the general health of humans who withstands exogenic and endogenic stimuli and damage via immune cells and related signaling pathways. Various diseases develop along with immune system dysfunctions. In steady‐state conditions, myeloid precursors in the bone marrow eventually differentiate into monocytes and neutrocytes, maintaining a normal and well‐balanced immune status. However, under some pathological processes, such as tumor progression, myeloid cells can abnormally differentiate into immature cells named myeloid‐derived suppressor cells (MDSCs).^1^

MDSC, a kind of cell that mainly accumulates in a few situations, such as cancer and chronic inflammations,^2^ plays important roles in immunosuppression, angiogenesis, tumor drug resistance, and tumor metastasis in the tumor microenvironment (TME).^3^ MDSCs can be simply classified into two types based on their precursor cells’ features:granulocytic/polymorphonuclear MDSCs (PMN‐MDSCs) and monocytic MDSCs (M‐MDSCs).^4^ While they share some molecular features and most functions, these two cell types also have their unique properties. For example, *CD14* and *CD15* expression differs between these types, which is a key feature for distinguishing them.^5^

In tumor progression, MDSCs can be generated and activated via complex molecular mechanisms and then recruited into the TME to perform protumoral functions, which is modulated mainly by CXC motif chemokine receptor (CXCR) 2.^6^ These cells primarily play immunosuppressive roles by affecting T cells, including suppressing T cell functions and promoting their apoptosis.^6^, ^7^ In addition, MDSCs also express immunosuppressive and trophic factors, including vascular endothelial growth factor (VEGF), interleukin (IL) 1/2/10, transforming growth factor‐β (TGF‐β), programmed death‐ligand (PD‐L) 1/2, indoleamine 2,3‐dioxygenase (IDO), inducible nitric oxide synthase (iNOS), and arginase‐1 (ARG‐1), to assist in immunosuppression, angiogenesis, and tumor progression.^6^

Since MDSCs have multiple mechanisms promoting tumor progression, MDSC‐targeted therapy appears to be a promising treatment for malignant tumors. Another notable peculiarity of MDSCs is that they are present in small quantities under healthy and physiological conditions. Therefore, therapy targeting MDSCs may reduce damage to normal tissues, side effects are expected to be averted.^1^ In recent, MDSC‐related targeted therapy has become an anticipated direction. Based on molecular structures, MDSC‐targeted drug therapies can be categorized into three groups: monoclonal antibodies (mAbs), small‐molecular inhibitors, and peptides. Some of these drugs have already been approved for clinical use in malignant tumors. For example, sunitinib, a receptor tyrosine kinase inhibitor (TKI), has been reported to deplete MDSCs in both peripheral blood and tumor sites.^8^, ^9^ mAbs with common target sites (e.g., programmed cell death 1 [PDCD1/PD‐1]/PD‐L1, cytotoxic T‐lymphocyte antigen 4 [CTLA‐4]) also negatively regulate MDSCs.^10^, ^11^, ^12^ In addition, with their high biocompatibility and convenience, tumor‐targeting peptides have become one of the most advanced and promising areas in tumor‐targeted therapies. These peptides can either suppress tumors directly or function as accurate carriers for antitumor drugs by recognizing and combining specific receptors; some also show anti‐MDSC effects.^13^

This review first presents the essential characteristics of MDSCs to briefly introduce them. Then, it describes the functional features of MDSCs in various conditions and classifies them based on their molecular mechanisms. This review mainly highlights the environmental specificity of MDSC, including the differences between tumor‐infiltrating and circulating MDSCs and between MDSCs from patients with cancer and healthy donors. The following section summarizes and emphasizes MDSC‐targeted drugs and therapies, hoping to provide researchers and clinical workers with guidance and insights into a new target in cancer therapy.

## 2 FEATURES OF MDSCs

### 2.1 Classification of MDSCs

In the bone marrow, myeloid cells can be briefly classified into two groups (granulocytes and mononuclear cells), which primarily contribute to the body's immune reaction. MDSCs are pathologically and immaturely differentiated from myeloid precursors (granulocytic and monocytic cell lines) and are divided into the two main populations as mentioned above (PMN‐MDSCs and M‐MDSCs).^4^ In particular, the phenotypic characteristics of these two MDSC types are fundamentally analogous to those of neutrophils and monocytes, respectively.^14^ Furthermore, a small proportion of myeloid precursors equipped with MDSC characteristics are considered early MDSCs.^15^

### 2.2 Origination and maturation of MDSCs

Myeloid cells in the bone marrow differentiate into mature neutrophils and monocytes in response to acute inflammatory stimulations, such as pathogenic invasion. Those differentiating into monocytes subsequently generate macrophages and dendritic cells (DCs) to provide protection and manifest a proinflammatory activity.^16^ However, these myeloid cells are observed to be activated immaturely in chronic pathological states, generating MDSCs.^17^, ^18^ Myeloid cell activation signals differ in the development of different pathogenic states. Persistent stimuli‐related myeloid cell activations are characterized by long‐lasting signals, comprising growth factors and inflammatory cytokines.^1^

MDSC accumulation from myeloid progenitor cells in vivo comprises two stages: immature myeloid cell expansion and pathological activation.^19^ The first stage is regulated by the granulocyte‐macrophage colony‐stimulating factor (GM‐CSF), granulocyte colony‐stimulating factor (G‐CSF), macrophage colony‐stimulating factor, stem cell factor, and some other chronic inflammatory or tumor‐related factors. These molecules induce expansion via signal transducer and activator of transcription (STAT) 3, interferon regulatory factor 8 (IRF8), CCAAT enhancer‐binding protein beta (CEBPB), retinoblastoma 1 (RB1), and Notch pathways.^20^ Various studies have shown the essential role of STAT3—an inducer of immunosuppressive regulation during MDSC expansion.^21^ However, STAT3 is only the regulatory factor of PMN‐MDSCs. While the population decline of PMN‐MDSCs is strongly correlated with the inhibition of STAT3 in the TME, the accumulation of M‐MDSCs is not relevant to the state of STAT3.^22^

In the second stage, besides inflammation‐promoting factors, such as IL‐1β and tumor necrosis factor (TNF)‐α, and some other mediators, MDSCs can also be activated by a specific highly conserved mechanism—endoplasmic reticulum stress.^20^ Most cells, including malignant cells, can barely survive under misfolded protein accumulating in the endoplasmic reticulum, mainly due to lower pH, higher free levels of radicals, and hypoxia in the TME. However, the unfolded protein response can be subsequently activated in MDSCs, as shown by the activation of inositol‐requiring enzyme 1, pancreatic endoplasmic reticulum kinase, and activating transcription factor 6.^23^, ^24^ The cascade of reactions induced by these molecules can promote MDSC differentiation, accumulation, and functioning,^23^ partly reversing the inhibitory effect of the adverse environment on tumor progression.

The immature differentiation of granulocytes in the bone marrow usually leads to the generation of PMN‐MDSCs. However, Mastio et al. showed that a branch of cells from the monocytic lineage could differentiate into PMN‐MDSCs.^25^ These precursors are named “monocyte‐like precursors of granulocytes” and can be detected only in the presence of a tumor. The downregulation of RB1 regulates the generation of this specific branch of myeloid cells.^25^ IRF8, which promotes granulocyte‐derived PMN‐MDSC expansion, barely affects the expansion of monocyte‐like granulocyte precursors.^25^ However, additional features of these monocyte‐derived PMN‐MDSCs must be elucidated.

### 2.3 Phenotypic characteristics of MDSCs

Different MDSC subtypes in mice share the same molecular features as CD11b^+^ Gr‐1^+^. Movahedi et al.^26^ first distinguished and purified M‐MDSCs and PMN‐MDSCs using the marker of Ly6G maker. Further studies refined the features of MDSCs in detail, showing that they have entirely different characteristics in mice and humans, with the subpopulation of MDSCs showing much heterogeneity. PMN‐MDSCs are described as CD11b^+^ Ly6G^+^Ly6C^low^ and M‐MDSCs as CD11b^+^ Ly6G^−^ Ly6C^high^ ^27^ in mice. And CD49d expresses mainly in murine M‐MDSCs rather than murine PMN‐MDSCs.^28^ The Gr‐1 molecule does not exist in humans. In humans, PMN‐MDSCs and M‐MDSCs are both CD11b^+^CD33^+^, but have discrepant CD14 and CD15: while PMN‐MDSCs and neutrophils are CD11b^+^CD14^−^CD15^+^ CD66b^+^ CD33^+^, M‐MDSCs and monocytes are CD11b^+^CD14^+^CD15^−^ CD66b^−^ CD33^+ 5^. However, density gradient is a distinguishing indicator of PMN‐MDSCs and neutrophils,^5^ and M‐MDSCs and monocytes can be distinguished by the status of major histocompatibility complex (MHC) class II molecule expression (i.e. human leukocyte antigen [HLA]‐DR).^29^

**FIGURE 1:**
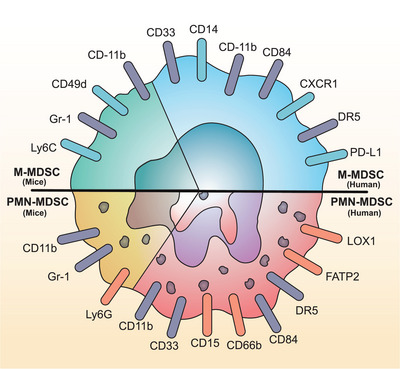
Main surface markers of monocytic myeloid‐derived suppressor cells (M‐MDSCs) and polymorphonuclear myeloid‐derived suppressor cells (PMN‐MDSCs) in mice and human. The green area represents mouse M‐MDSCs, the yellow area represents mouse PMN‐MDSCs, the blue area represents human M‐MDSCs, and the red area represents human PMN‐MDSCs. The gray surface markers represent the shared markers of both MDSC subtypes; the blue ones represent the marker only shown in M‐MDSCs; while the orange ones are indicated as the unique markers of PMN‐MDSCs.

In the TME, more features of MDSCs with specificity have been identified. Lectin‐like oxidized low‐density lipoprotein receptor 1 (LOX1), a kind of transmembrane glycoprotein with a mass of 50 kDa, is defined as a specific marker of PMN‐MDSCs,^30^, ^31^ and another specific marker is fatty acid transport protein 2 (FATP2).^32^ PD‐L1 is primarily expressed in M‐MDSCs, functioning as one primary approach for inducing immune regulation.^3^ In addition, a single‐cell transcriptomics analysis showed that CD84^+^ cells in tumor tissues could be marked as MDSCs.^33^ Highly specific molecules can function as markers of MDSCs and serve as promising targets for cancer immunotherapy (Figure 1). Several other literatures have also reviewed the surface markers of human and murine MDSCs in detail,^4^, ^34^ providing references of high‐quality for MDSC distinguishment.

Notably, the current definition and classification methods for MDSC subsets are based on the immune suppressive functions and surface markers. However, MDSCs have more complex heterogeneity and plasticity, and a more integrated definition and classification of MDSCs should focus on the differences in immunosuppressive mechanisms (e.g., stimulating factors and impacted pathways/cells).^35^

## 3 ENVIRONMENTAL SPECIFICITY OF MDSCs

As previously mentioned, MDSCs rarely exist under healthy conditions in humans. Under tumor progression, MDSCs can form during tumor progression, leading to their expansion and discrepant quality. Therefore, it is essential to find out how MDSCs differ in different sites so that they can be targeted more accurately. Since the differences between mature myeloid cells and MDSCs and between MDSC subpopulations have already been detailed in other reviews,^4^, ^35^ our review focuses on the discrepancies in environmental factors that arise.

### 3.1 Differences between MDSCs from patients with cancer and healthy donors

**FIGURE 2:**
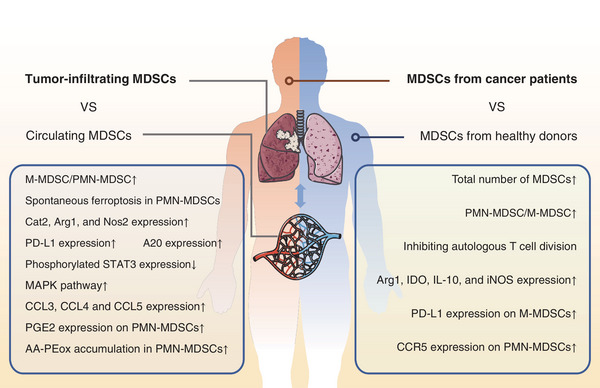
The differences of myeloid‐derived suppressor cells (MDSCs) under various environments. The descriptions in the left frame demonstrate the features of tumor‐infiltrating MDSCs compared with circulating MDSCs. The descriptions in the right frame display the features of MDSCs from cancer patients compared with MDSCs from healthy donors. Cat2, cationic amino acid transporter 2; Arg‐1, arginase‐1; Nos2, nitric oxide synthase 2; PD‐L1, programmed death ligand 1; STAT3, signal transducer and activator of transcription 3; MAPK, mitogen‐activated protein kinase; CCL, C‐C motif chemokine ligand; IDO, indoleamine 2,3‐dioxygenase; IL‐10, interleukin‐10; iNOS, inducible nitric oxide synthase; CCR, C‐C motif chemokine receptor; PGE2, prostaglandin E2; AA‐PEox, arachidonic acid‐poly (ethylene oxide).

The number of MDSCs was significantly larger in patients with cancer than in healthy donors.^36^, ^37^ The proportion of circulating PMN‐MDSCs is larger in most patients with cancer than that in healthy individuals, indicating that the largest expansion occurs in PMN‐MDSCs.^38^ The expression of LOX1 in PMN‐MDSCs and PD‐L1 in M‐MDSCs is not significantly higher in patients with tumors than in healthy populations,^38^ and the quantity of MDSCs is closely related to the clinical cancer stage and tumor metastasis in colorectal carcinoma.^37^ These findings indicate that the number of MDSCs is associated with the strength of immunosuppressive activities. Regarding immunosuppression, Zhang et al.^37^ found that MDSCs from healthy donors could not inhibit the division of autologous T cells, unlike those from patients with tumors. MDSCs from patients with cancer express higher *ARG‐1, IDO, IL10*, and *iNOS* expression was higher in MDSCs from patients with cancer than from healthy donors.^39^, ^40^ While M‐MDSCs produced more PD‐L1, PMN‐MDSCs produced more C‐C motif chemokine receptor 5 (CCR5).^41^ Briefly, the transition from a steady state to cancerous state results in MDSC expansion and the enhancement of their immunosuppressive functions (Figure 2).

### 3.2 Differences between tumor‐infiltrating and circulating MDSCs

#### 3.2.1 Differences in molecular phenotype

MDSCs primarily form in the bone marrow but can also be induced by tumor‐related cytokines in peripheral blood from peripheral blood myeloid cells. The phenotypic characteristics of MDSCs vary in the tumor site and peripheral blood in patients with different tumors. In patients with non‐small‐cell lung cancer (NSCLC), PMN‐MDSCs obtained from tumor tissue were CD11b^+^HLA^−^DR^−/low^CD14^−^CD15^+^,^41^ while ILT3^high^ can be acquired from peripheral blood.^42^ Similarly, M‐MDSCs in NSCLC sites are CD11b^+^ HLA‐DR^−/low^ CD14^+^ CD15^−^ ^41^; however, circulating M‐MDSCs can be featured as CD16^low^.^43^ Discrepancies also occur in other tumors,^37^ although many studies have found identical MDSC phenotypes in tumor tissue and peripheral blood.^44^, ^45^ Therefore, the phenotypes of MDSCs can vary in different tissues; however, resemblance among different tumors could barely be noted, partly because the phenotypes of MDSCs in different studies are diverse.

#### 3.2.2 Differences in quantity and subtype proportion

In some cancer types, there are more tumor‐infiltrating MDSCs than MDSCs in peripheral organs.^41^, ^46^ Since M‐MDSCs have stronger immunosuppressive activities, the proportion of these cells in total MDSCs has gained increasing attention. In tumors, PMN‐MDSCs predominate the circulating MDSC population, while the M‐MDSC/PMN‐MDSC ratio increases in the TME (Figure 2).^47^, ^48^

#### 3.2.3 Differences in molecular expression and function

Tumor‐infiltrating MDSCs have stronger immunosuppressive activities than circulating MDSCs. This difference can be attributed to the functional differences in the total number of MDSCs and the M‐MDSC/PMN‐MDSC ratio. Furthermore, on a per‐cell basis, tumor‐infiltrating MDSCs are more likely to be a suppressor. MDSCs in prostate cancer tissues can upregulate cationic amino acid transporter 2 (*CAT2*), *ARG‐1*, and nitric oxide synthase 2 (*NOS2*) expression, decreasing l‐arginine production, increasing nitric oxide (NO) production, and enhancing the detrimental effects on T cells.^49^ Hypoxia‐inducible factor 1 subunit alpha (*HIF1α*) expression increases in the tumor environment due to hypoxia, enhancing PD‐L1 expression in MDSCs.^41^ Kumar et al.^50^ considered phosphorylated STAT3 the key factor inducing MDSCs to differentiate into tumor‐associated macrophages (TAMs), reporting that tumor‐infiltrating MDSCs had a lower phosphorylated STAT3 level to retain their immature state. Our previous study noted higher basal mRNA levels and protein levels of TNF‐alpha‐induced protein 3 (TNFAIP3/A20) increased in tumor‐infiltrating MDSCs than in MDSCs from the spleen.^51^ We also observed an upregulation of the mitogen‐activated protein kinase (MAPK) pathway in tumor‐infiltrating MDSCs, especially the extracellular signal‐regulated protein kinase (ERK)‐related pathway.^47^ Tumor‐infiltrating M‐MDSCs in lymphoma and melanoma can induce high C‐C motif chemokine ligand (*CCL*) 3, *CCL4*, and *CCL5* expression to recruit regulatory T cells (Tregs) via chemokine–CCR5 interactions (Figure 2).^52^

**FIGURE 3:**
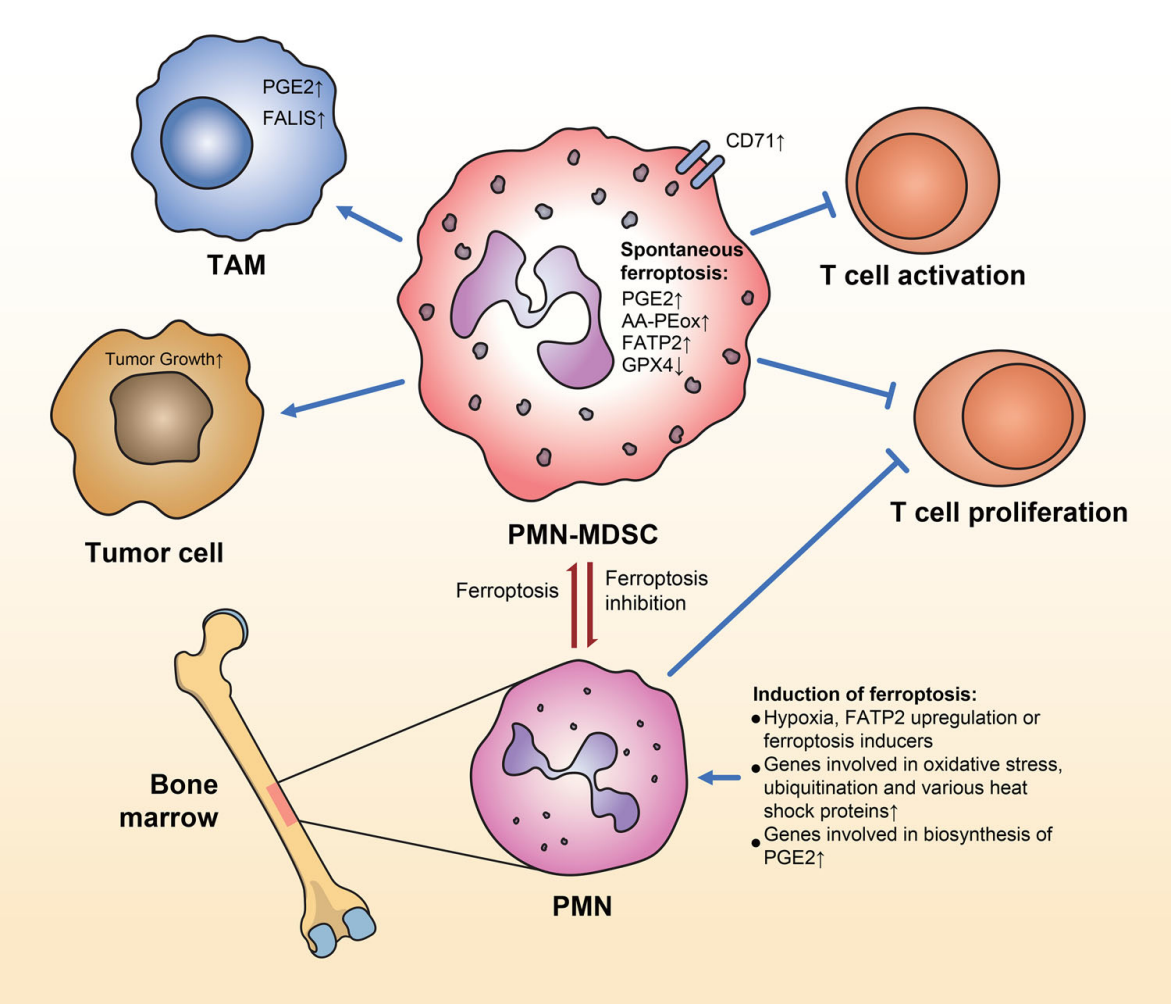
The immunosuppressive effects of ferroptosis in tumor‐infiltrating polymorphonuclear myeloid‐derived suppressor cells (PMN‐MDSCs). Ferroptosis occurs spontaneously in tumor‐infiltrating PMN‐MDSCs. When classical PMNs are influenced by hypoxia, FATP2 upregulation, or ferroptosis inducers, they may transform to PMN‐MDSCs and obtain the ability of immunosuppression. Tumor‐infiltrating PMN‐MDSCs were observed upregulating ferroptosis feature genes, gathering AA‐PEox, PGE2, and FATP2. The downregulation of GPX4 is also observed, which restrains the proliferation and activation of CD8^+^ T cells. In addition, TAMs are activated with the upregulation of the expressions of FALIS and PGE2. PGE2, prostaglandin E2; FALIS, ferroptosis‐associated lipid signals; TAM, tumor‐associated macrophage; AA‐PEox, arachidonic acid‐poly (ethylene oxide); FATP2, fatty acid transport protein 2; GPX4, glutathione peroxidase 4.

Tumor‐infiltrating PMN‐MDSCs but not M‐MDSCs or circulating PMN‐MDSCs were recently found to induce spontaneous ferroptosis, one basis of their immunosuppressive ability. Ferroptosis is the dominating cell death pattern in tumor‐infiltrating PMN‐MDSCs, causing tumor progression.^53^ Unlike circulating PMN‐MDSCs, tumor‐infiltrating PMN‐MDSCs induced the upregulation of ferroptosis‐related genes and the accumulation of oxidized phosphatidylethanolamine containing arachidonic acid, one of the main characteristics of ferroptosis, and prostaglandin E2 (PGE2). The induction of ferroptosis, which may partly underlie the downregulation of hypoxia‐related glutathione peroxidase 4 (GPX4) in the TME, gives polymorphonuclear cells immunosuppressive activity, restraining the proliferation of CD8^+^ T cells. Consequently, ferroptosis convert polymorphonuclear cells into PMN‐MDSCs. Conversely, suppressing ferroptosis in tumor‐infiltrating PMN‐MDSCs activates antitumor immunity, such as neutrophil‐related immunity and antigen processing/presentation, which turns PMN‐MDSCs back into classical polymorphonuclear cells.^53^ Ferroptosis inhibition in tumor‐infiltrating PMN‐MDSCs downregulates ferroptosis‐associated lipid signals (*FALIS*) and *PGE2* expression in TAMs, leading to their dysfunction. Substantial increases occur in memory and effector CD8^+^ T cells and natural killer (NK) cells due to upregulation of IL‐2‐ and TNF‐α‐associated inflammatory signaling and suppression of oxidative phosphorylation.^53^ Eventually, ferroptosis inhibition results in the regeneration of antitumor immunity (Figure 3).

### 3.3 Differences between artificially induced circulating MDSCs and noninduced circulating MDSCs

Some studies have artificially induced circulating MDSCs using cytokines, such as GM‐CSF, IL‐1, IL‐6, VEGF, TNF‐α, and PGE2, which differ from noninduced MDSCs.^54^ Compared with noninduced circulating M‐MDSCs, cytokine‐induced M‐MDSCs have low expression of S100 calcium‐binding protein A9 (*S100A9*), a chemokine strategic for MDSC migration,^55^ but demonstrate good performance against T cell proliferation.^56^ Generated peripheral blood MDSCs have various immunosuppressive activities via different inducing factors; processing peripheral blood myeloid cells with the combination of GM‐CSF and IL‐6 is one of the most effective ways. In Roussel's study, the proportion of MDSCs increased more than fivefold after induction,^55^ and in Lechner's study, the generated MDSCs inhibited T cell proliferation by 80%.^54^ Their increased immunosuppressive activity was closely associated with the upregulation of iNOS, NADPH oxidase 2 (NOX2), VEGF, TGF‐β, and PD‐L1.^54^

### 3.4 Differences between MDSCs from patients with tumors and premalignant conditions

Circulating MDSCs in patients with cancer and premalignant conditions share the same composition and function but with larger amounts in cancer environments.^57^ This finding suggests that MDSCs in both cancerous and precancerous lesions can be targeted using similar drugs. T cell proliferation and interferon‐γ (IFN‐γ) production return to normal after MDSC elimination in precancerous,^57^, ^58^ but not in cancerous environments.^59^ Therefore, targeting MDSCs in precancerous lesions is an effective way to reduce cancer risk.

## 4 FUNCTIONS OF MDSCs

MDSCs play a vital immunosuppressive role in various pathological processes, especially in all kinds of tumors and TMEs. Herein, we orderly introduce the specific functions of MDSCs in a physiological situation, inflammation, and tumor progression.

### 4.1 Functions of MDSCs in healthy conditions and inflammation

#### 4.1.1 Functions of MDSCs in healthy conditions

Bone marrow‐derived myeloid cells usually differentiate into mononuclear cells and granulocytes.^60^ With their small quantity and modest functions, MDSCs rarely show immunosuppressive effects under physiological homeostasis conditions. However, there are exceptions: pregnancy and neonates. Since a fetus is a type of allograft in a mother, preventing its rejection, or maternal‐fetal tolerance, is vital.^61^, ^62^ One study observed SR‐E1^+^ PMN‐MDSCs in all stages of pregnancy, which correlated negatively with intrauterine growth retardation.^63^ In this process, MDSCs inhibit T cell, T helper cell, and NK cell function by producing reactive oxygen species (ROS), expressing *ARG‐1*, or functioning through cell contact. Moreover, MDSCs appear in neonates for a transient period.^64^ These cells may originate from cord blood^65^ or be transported via breast milk^66^ and participate actively in inflammation regulation.^67^

#### 4.1.2 Functions of MDSCs in infections

The roles of MDSCs in chronic and acute infectious diseases have been elucidated in recent years, whose immunosuppressive ability promotes the progression of these infectious diseases, and the detailed mechanisms of MDSCs acting on individual diseases were summarized by several reviews.^68^, ^69^, ^70^ In brief, to enhance the infectious effects, MDSCs commonly produce immunosuppressive metabolites via iNOS and ARG‐1, and increase the expressions of immunosuppressive molecules like IL‐10, ROS, STAT3, and so on, mainly to prevent immune cell proliferation and activation, especially T cells.^71^, ^72^, ^73^, ^74^, ^75^ For instance, in the chronic infectious condition induced by *Staphylococcus aureus* (*S. aureus*), a large number of MDSCs were found in the surrounding bacterial biofilm, contributing to the persistence and low clearance of *S. aureus*, by secret iNOS, ARG‐1, as well as IL‐10 to inhibit the infiltration of immune cells^76^; in turn, stimulated by MDSCs, *S. aureus* will upregulate formate acetyltransferase (pflB) to form a larger and more pervasive biofilm.^77^ Apart from the common mediatory mechanisms, MDSC can induce high‐level ER stress to enhance its immunosuppressive activity, therefore leading to the promotion of leprosy and tuberculosis.^78^, ^79^ During sepsis or some parasite infections like *Schistosoma japonicum*, MDSCs are also able to increase the expression of PD‐L1, modulating the immunoactivity of T cells via the PD‐1/PD‐L1 pathway.^80^, ^81^

In virus infections, since the acquired immune deficiency syndrome is characterized by immune deficiency, the role of MDSCs after human immunodeficiency virus (HIV) invasion has been widely explored. For one thing, MDSCs will inhibit the proliferation of CD4^+^ and CD8^+^ T cells while promoting the expansion of Tregs and HIV infection.^82^, ^83^ For another, however, they are beneficial to inhibit the persistent immune activation and the accompanying chronic inflammations induced by HIVs.^84^ Similarly, in severe COVID‐19 patients, IL‐8 will induce the transitory recruitment of PMN‐MDSCs in peripheral blood to help the patients’ recovery^85^ to fight against hyperinflammation. Therefore, the immunosuppressive activity of MDSCs can also have positive effects during virus infections.

#### 4.1.3 Functions of MDSCs in autoimmune diseases

Rheumatoid arthritis (RA) is a systemic, cartilage‐and‐bone‐destructive autoimmune disease of which immune cell infiltration and accumulation in the synovial joints have a vital role in the pathogenesis,^86^, ^87^, ^88^ and MDSCs possess immunosuppressive functions to alleviate the worsening of RA. MDSC accumulations were found in synovial fluid, lymph nodes, peripheral blood, as well as spleen of RA patients.^89^, ^90^, ^91^ They inhibit CD4^+^ T cell proliferation (via ROS, NO, IFN‐γ generation, etc.) and infiltration and the proinflammatory cytokine production by CD4^+^ T cells, restrict B cell proliferation and antibody production (via NO, PGE2 production), promote Treg cell proliferation (via IL‐10 generation), as well as stimulate IL‐10^+^ Breg cell generation (via exosomal PGE2 secretion).^90^, ^92^, ^93^, ^94^, ^95^ Although MDSCs display obvious anti‐inflammatory capacities in autoimmune diseases, they also surprisingly play some proinflammatory roles.^90^, ^91^ In RA, with the progression of disease, the number of MDSCs also expands, which is negatively related to the number of T helper 17 cells in peripheral blood.^96^, ^97^ The research found that MDSCs could stimulate T helper 17 cell differentiation by producing IL‐1β, reprograming T helper 17 cells into pro‐osteoclastogenic phenotype, promoting osteoclast differentiation to aggravate RA severity.^97^, ^98^, ^99^ This kind of uncharacteristic, proinflammatory effect of MDSCs has been also demonstrated in other autoimmune diseases.^100^ The abnormal activation of Notch1 signaling plays an important role in the pathogenesis of systemic lupus erythematosus (SLE), which also promote the MDSC differentiation process.^101^ In the peripheral blood of SLE patients, the frequency of MDSCs increase with the disease severity, and MDSCs promote T helper 17 cell differentiation via the activation of *ARG‐1*, contributing to SLE‐related renal injury.^100^ The activation of ARG‐1/miR‐322‐5p/TGF‐β pathway can also change the T helper 17 cell/Treg cell ratio in SLE.^102^ In experimental autoimmune encephalomyelitis (EAE), similarly, spleen‐derived MDSCs facilitate T helper 17 cell differentiation in an IL‐1β‐dependent manner,^103^ while lung‐derived MDSCs generate IL‐6 to achieve the same purpose.^104^ In brief, MDSC is a double‐edged sword during autoimmune disease progression, but its immunomodulatory mechanisms in different stages of disease still need further exploration. Apart from its function of affecting immune microenvironment, MDSC was discovered to promote oligodendrocyte precursor cell maturation and remyelination by secreting osteopontin in EAE models,^105^ indicating that MDSC can not only be regarded as an immune cell but also has potential in neural system regeneration.

#### 4.1.4 Functions of MDSCs in organ transplantation

As a kind of therapeutic tool, MDSCs can be used for immunosuppression in organ transplantation for beneficial utilization. Acute graft‐versus‐host disease (GVHD) is the most substantial threat among organ transplant recipients, and MDSCs play a positive role in transplantation immune tolerance, reducing the incidence of GVHD.^106^, ^107^, ^108^ Tobias et al.^109^ found that the level of PMN‐MDSCs increases in stable lung transplant recipients (52.1% when that of the control group reaches 9.4%) compared with that in patients with infection (28.2%) and chronic lung allograft dysfunction (33.0%). Okano et al.^110^ added MDSCs in a coculture with donor‐reactive T cells and donor intestinal epithelium, achieving an increased organ survival rate. Another study among 38 kidney transplant patients concluded that the level of M‐MDSCs increased at 6 and 12 months after transplantation.^111^

#### 4.1.5 Functions of MDSCs in anti‐inflammatory therapy

Another possible direction is to apply MDSCs in anti‐inflammatory therapy. Various substances can induce MDSC production in vivo or in vitro (Table 1). Xu et al.^112^ identified mitogen‐activated protein kinase kinase kinase 8 (MAP3K8/Tpl2), a protein kinase acting as a pivotal mediator of MDSC recruitment in fulminant hepatitis. When patients develop fulminant hepatitis, their IL‐25 level increases, activating the signal pathway mediated by IL‐17 receptors A/B (IL‐17RA/RB) and Tpl2, leading to the expression of CXC motif chemokine ligands (CXCLs) 1/2. Then, the increased CXCL1/2 expression stimulates the recruitment of MDSCs, promoting inflammation inhibition. Another study showed that artepillin C in green propolis attenuates allergic asthma by inducing M‐MDSCs.^113^ Studies have found an augmented frequency of MDSCs in both in vitro and in vivo experiments. Moreover, artepillin C‐treated mice had a lower frequency of eosinophils in their bronchoalveolar lavage fluid, lower lung inflammation score, and lower mucus score. Propolis therapy may also positively affect PMN‐MDSC recruitment.^114^ However, further research on the exact mechanism is needed. In heart failure models, MDSCs also participated in myocardial protection. In a recent study, pharmaceutical depletion of MDSCs significantly increased inflammation and pathological cardiac remodeling.^115^

**TABLE 1:** Different substance inducing myeloid‐derived suppressor cells (MDSCs) in various models.

Study	Year	In vivo or in vitro	Substance inducing MDSC	Model	References
Lechner et al.	2010	In vitro	IL‐6	Healthy donor peripheral blood mononuclear cell	^54^
	2010	In vitro	GM‐CSF	Healthy donor peripheral blood mononuclear cell	
Morales et al.	2010	In vitro	GM‐CSF	Murine bone marrow CD11b‐Gr1‐ cells	^116^
Chen et al.	2013	In vitro	GM‐CSF, IL‐4, and CpG oligodeoxynucleotide	Murine bone marrow cell	^117^
Liu et al.	2015	In vitro	IL‐6, GM‐CSF, and norepinephrine	Healthy donor peripheral blood mononuclear cell	^118^
Yang et al.	2019	In vitro	M‐CSF and IFN‐γ	Murine bone marrow cell	^119^
Scheurer et al.	2021	In vitro	GM‐CSF	Murine bone marrow cell	^120^
Wang et al.	2021	In vitro	M‐CSF and phorbol 12‐myristate 13‐acetate	Murine bone marrow cell	^121^
Sarra et al.	2013	In vivo	IL‐25	d‐Gal/lipopolysaccharide (LPS) induced fulminant hepatitis mice	^122^
Zhou et al.	2018	In vivo	Rapamycin	Heart failure patients; isoproterenol infusion/transverse aortic constriction mice; induced heart failure mice	^115^
Xu et al.	2019	In vivo	Tpl2	Propionibacterium acnes/LPS induced fulminant hepatitis mice	^112^
Martins et al.	2021	In vivo and in vitro	Artepillin C	In vitro M‐MDSC culture; Ovalbumin induced asthma mice	^113^

**FIGURE 4:**
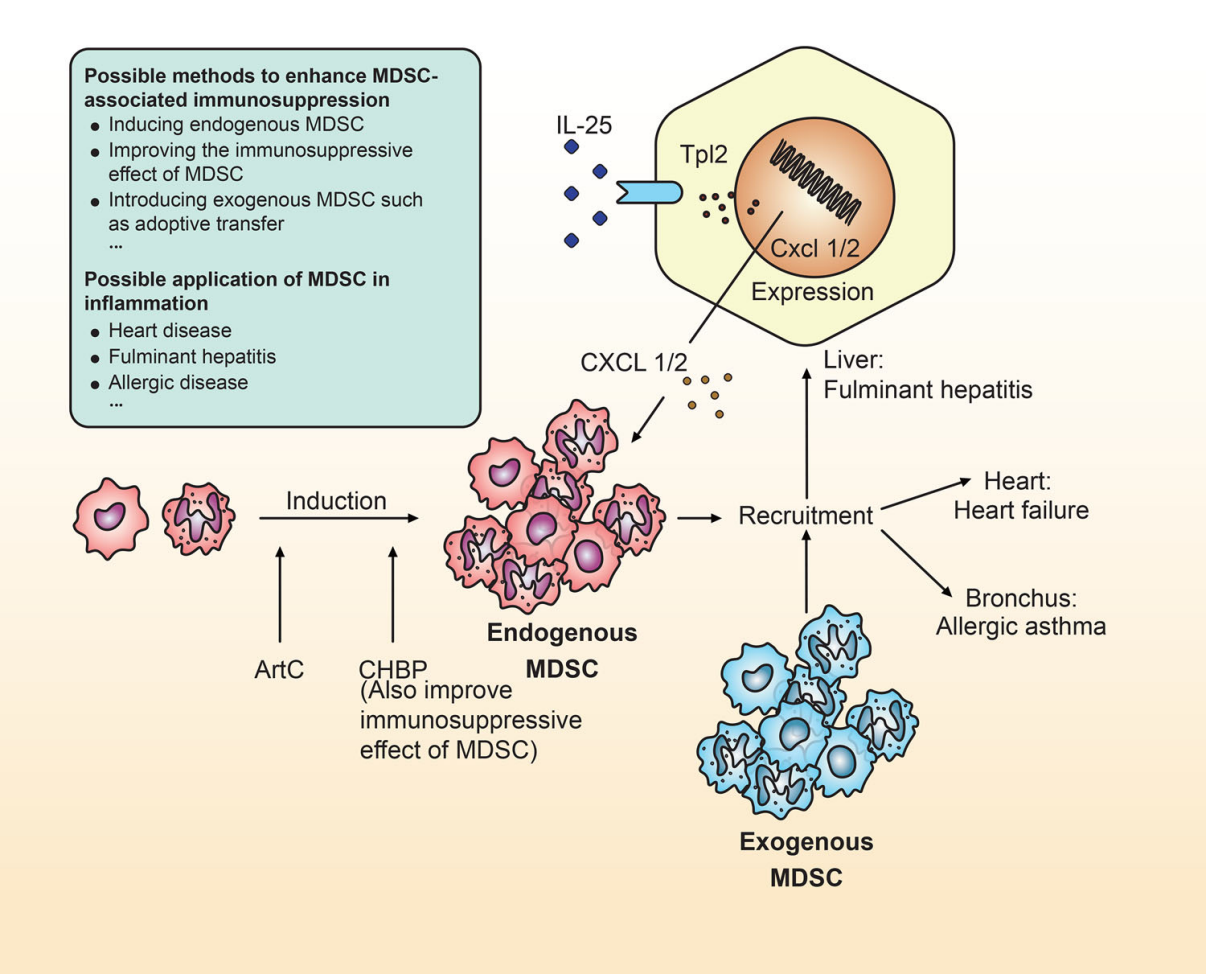
Possible methods to enhance myeloid‐derived suppressor cell (MDSC)‐associated immunosuppression and the application of MDSC in anti‐inflammation therapy. ArtC, artepillin C; CHBP, cyclic helix B peptide; IL‐25, interleukin‐25; Tpl2, Tumor progression locus 2; CXCL, CXC motif chemokine ligand.

As mentioned above, MDSCs can be used when treating inflammation‐related diseases in multiple organs in three ways: first, endogenous MDSCs are able to be increased by inducing their formation or inhibiting continued differentiation. For example, rapamycin can inhibit MDSC differentiation and improve heart failure.^115^ Second, the immunosuppressive effects of MDSCs are expected to be improved. For example, cyclic helix B peptide can induce IL 7 receptor (ILR7/CD127)^+^ M‐MDSCs with a stronger immunosuppressive activity.^123^ Third, exogenous MDSCs can be introduced via adoptive transfer, which has been proposed in organ transplantation; this method may also be applied in treating inflammatory diseases (Figure 4).^124^ There are currently fewer studies in this area, and there may be a broad scope for development.

### 4.2 Tumor‐related immunosuppressive functions of MDSCs

#### 4.2.1 Effects of MDSCs on T cells

The role of MDSCs in the TME mainly involves inhibiting of T cell activation. The direct effects of MDSCs are principally to reduce the expression of specific recognition receptors on the T cells’ surface and to promote T cell apoptosis. Their indirect effect is to regulate the T cells’ proliferation and differentiation by changing the microenvironment's physical and chemical components.

##### Direct effects on T cells

MDSCs can produce large amounts of ROS and reactive nitrogen species (RNS), which inhibit the activation of the Janus kinase (JAK) 3/STAT5 signaling pathway, downregulating the expression of MHC‐II molecules and inhibiting T cell apoptosis.^118^ Meanwhile, ROS can also induce T cells to express Fas ligand (FASLG/FasL), which contributes to the activation of T cell apoptosis through a Fas cell surface death receptor (Fas)–FasL interaction.^125^ PMN‐MDSCs mainly produce ROS and function by directly contacting T cells.^126^ M‐MDSCs mainly produce RNS, impacting T‐cell apoptosis without directly contacting them since RNS are more stable than ROS.^127^ Mionectic TME‐generated HIF‐1α can also promote PD‐L1 expression on the surface of PMN‐MDSCs, leading to T cell apoptosis.^128^

Besides their impact on the death of proinflammatory T cells, MDSCs can also directly mediate T cells’ generation, differentiation, and migration of T cells. MDSCs inhibit the generation and proliferation of tumor antigen‐specific T helper 1 cells by producing IL‐6.^129^ They also promote the differentiation of CD4^+^ T cells into Tregs by releasing of cytokines or cell‐to‐cell contact and Treg proliferation.^7^ For example, through the TGF‐β1‐dependent pathways, MDSCs promote the generation of Tregs, and *IDO* overexpression by MDSCs causes Tregs migrate to tumor sites and lymph nodes.^130^ Moreover, the NO produced by M‐MDSCs can react with superoxide to form peroxynitrite (ONOO–), which nitrifies T‐cell receptors and T‐cell‐specific chemokines to directly block T‐cell migration.^131^ Zhao et al.^132^ discovered that MDSCs could release the specific metabolite itaconate, derived from the tricarboxylic acid cycle, to repress the proliferation and the antitumor cytotoxicity of CD8^+^ T cells by decreasing aspartate, serine, and glycine synthesis and reducing the dominating aggressor granzyme and perforin. Itaconate can serve as a promising cancer therapy target and a tumor prognosis biomarker.

##### Indirect effects on T cells

M‐MDSCs can degrade arginine by secreting ARG‐1 and RNS. When arginine is competitively consumed in the microenvironment, the T‐cell‐receptor ξ chain and CD3 expression on T cells’ surface is downregulated, and the expression of cyclin D3 (CCND3) and cyclin‐dependent kinase 4 (CDK4) is inhibited, inducing T cell cycle arrest in the G0/G1 phase.^133^ In addition, T cells cannot synthesize cysteine autonomously and only use cysteine released into the environment. PMN‐MDSCs use solute carrier family 7 member 11 (SLC7A11) to scramble for cysteine with T cells by transferring it into the cytoplasm,^118^ inhibiting T cell activation, and promoting their apoptosis. Moreover, by producing IL‐1β, IL‐6, IL‐23, and PGE2, MDSCs can promote the differentiation and proliferation of T helper 17 cells.^134^ The production of IL‐17 by T helper 17 cells inhibits the antitumor effect of T cells.^135^ The specific immune checkpoint protein V‐domain immunoglobulin suppressor of T cell activation (VISTA) is crucial for regulating the effector functions of myeloid cells. By expressing VISTA, MDSCs can also participate in the negative regulation of T cell antitumor functions.^136^

#### 4.2.2 Effects of MDSCs on macrophages

The inhibitory effect of MDSCs on immune cells is also manifested in macrophages. MDSCs are involved in activating of TAMs and promoting the conversion of M1 to M2/TAM.^137^ They transform macrophages in tumor‐bearing individuals primarily by increasing IL‐10 production and decreasing macrophage‐derived IL‐12 production. Sinha et al.^138^ found that coculturing macrophages activated via lipopolysaccharide and IFN‐γ with MDSCs for 16 hours greatly downregulated their IL‐12 production. STAT3 has a unique function in controlling the differentiation of MDSCs into TAMs in the tumor environment.^50^

While TAMs and MDSCs are regarded as independent entities, the boundary between them is unclearly demarcated, and they have many common features.^139^ Tumor‐infiltrating MDSCs have the pleiotropic characteristics of M1 and M2 macrophages.^140^ M‐MDSC‐derived macrophages retain the main characteristics of their precursors, including immunosuppressive activity. S100A9 promotes the M2 polarization of macrophages to a certain extent.^140^ M‐MDSC‐derived macrophages retain *S100A9 expression*, but most monocyte‐derived macrophages or tissue‐resident macrophages do not. The difference in *S100A9* expression indicates that the presence of S100A9 in macrophages is critical to its inhibitory activity.

#### 4.2.3 Expression of immunosuppressive and trophic factors

MDSCs recruit suppressive immune cells by expressing TGF‐β, VEGF, fibroblast growth factor 2 (FGF2), MMP9, IL‐10, PD‐L1/2, IDO, iNOS, and ARG‐1 and establishing a microenvironment that suppresses host immunity by expressing suppressive cytokines (e.g., IL‐10 and TGF‐β) and surface molecules (e.g., PD‐L1 and PD‐L2).^141^

##### VEGF and MMP‐9

The nonimmunosuppressive functions of MDSCs include tumor angiogenesis and metastasis.^142^ MDSCs can express VEGF and basic FGF (*bFGF*) to help tumor cells build new vascular systems and deliver nutrients and oxygen to cancer cells.^143^ VEGF is a key angiogenic factor in tumors and participates in the initial stage of tumor development, progression, and metastasis.^144^ It stimulates vascular endothelial cells’ proliferation and survival, increases vascular permeability, and recruits vascular progenitor cells from the bone marrow, promoting tumor angiogenesis.^145^

PMN‐MDSCs can secrete proangiogenic factors.^143^ With their ability to activate the p38 MAPK and ERK1/ERK2 signaling pathways, PMN‐MDSCs can upregulate *VEGF* and *CXCR* expression in tumor cells,^146^ inducing proangiogenic effects and facilitating angiogenesis.^147^ Coinoculation of M‐MDSCs with tumor cells promoted TME microvessel formation and vascular leakage more than tumor cells alone.^148^

Notably, unlike the formation of blood vessels under physiological conditions, tumor blood vessel formation has a disorganized structure and a discontinuous and incomplete vessel wall.^55^ MDSCs secrete matrix metalloproteinases (MMPs), which degrade the extracellular matrix and thus promote angiogenesis.^149^ MMP‐9 activity is required to promote angiogenesis.^143^ It disrupts the basement membrane and alters the local microenvironment,^150^ promoting tumor progression and enabling tumor cells to cross the extracellular matrix into the peripheral circulation and become circulating tumor cells (CTCs). VEGF upregulates the activity of NK cells and their natural immune response and inhibits DC differentiation and development,^151^ suggesting its role in CTCs immune escape. While direct stimulatory effects of VEGFA have not been reported, the VEGFC–VEGF receptor (VEGFR) 3 axis has been reported to promote megakaryopoiesis and increase the platelet count, thereby further protecting CTCs.^144^

In addition, VEGF secreted by MDSCs directly effects on cancer cells or tumor stem cells. It may induce cancer cell proliferation and promote tumor‐initiated cell self‐renewal by activating VEGFR1 signaling.^145^ VEGF may also exert a proinflammatory cytokine release effect through VEGFR2/STAT3 signaling, inducing increased IL‐6, IL‐8/CXCL8, and growth‐regulated protein‐α/CXCL1 secretion by endothelial cells (nonleukocytes) and exerting an immunosuppressive effect.

##### TGF‐β

TGF‐β is secreted by various tumor cells and is closely associated with tumor growth and immunity.^152^ Inhibition of TGF‐β, PD‐L1, and LAIR‐1 was recently reported to effectively control the growth of collagen‐rich mouse MC38 colon cancer and EMT6 breast cancer, leading to tumor cure and long‐term tumor‐specific protection.^153^ In the TME, dysregulated TGF‐β signaling suppresses antitumor immunity and promotes cancer fibrosis, epithelial–mesenchymal transition (EMT), and angiogenesis. TGF‐β is an autocrine survival signal for myeloid precursor cells and drives their differentiation into highly immunosuppressive MDSCs at the expense of macrophages and DCs.^154^

MDSCs induce EMT in tumor cells via the TGF‐β signaling pathway. They can secrete multifunctional proteoglycans to induce EMT in cancer cells via the TGF‐β signaling pathway,^155^ promoting tumor invasion and metastasis.^156^ PMN‐MDSCs preferentially accumulate in primary tumors where they induce cancer cells to undergo EMT, facilitating the rapid acquisition of a mobile phenotype by cancer cells and leading to multinodal growth, systemic spread, and distant metastasis of the primary tumor.^157^ In the TME, dysregulated TGF‐β signaling suppresses antitumor immunity and promotes tumor fibrosis, EMT, and angiogenesis.^154^

TGF‐β is also involved in downregulating the expression of the killer cell lectin‐like receptor K1 (KLRK1/NKG2D) on the NK cell surface,^148^ inhibiting their production of cytokines, such as IFN‐γ, CCL5, and protein 1 alpha (MIP1α/CCL3).

##### IL‐10 and IL‐6

MDSCs induce the differentiation of regulatory B cells and regulatory DCs^158^ by secreting IL‐10. In addition, IL‐10 inhibits the activation and proliferation of NK cells, NK T cells, and DCs and the production of various antitumor cytokines (e.g., IL‐12),^159^ attenuating the immune response in the TME and facilitating tumor growth and spread of tumors. Tumor cell‐derived IL‐10 can promote autonomous cell growth and immune escape in diffuse large B cell lymphomas, and neutralizing IL‐10 signaling can reduce tumor growth.^160^ MDSCs secrete IL‐6, IL‐10, and IL‐23, which also inhibit the activation and proliferation of NK cells and NK T cells.^161^ Conversely, IL‐6 acts as a classical agonist to activate the JAK/STAT3 pathway and induce the expression of the MYC proto‐oncogene bHLH transcription factor (*MYC*/*c‐Myc*).^162^ The upregulation of STAT3 by IL‐6 also promotes *PD‐L1* and *ARG‐1* expression and NO and ROS production, increasing MDSC function.^163^ IL‐6 can also recruit circulating MDSCs into the TME.^164^

##### S100A8 and S100A9

MDSCs also secrete chemokines, such as S100 calcium‐binding proteins A8 (S100A8) and S100A9, which are actively transported to the TME and distant tissues via exosomes secreted by tumor‐associated immune cells (e.g., MDSCs).^55^ They further stimulate the release of inflammatory cytokines via Toll‐like receptor 4,^165^ promote Ca^2+^ influx, immune cell recruitment, and inflammatory processes^166^ and are involved in MDSC migration to tumor sites. After release, S100A8/A9 block the differentiation of MDSCs into DCs, leading to an increase in MDSCs,^150^ further promoting MDSC accumulation and thus coordinating the formation of an immunosuppressive microenvironment conducive to tumor growth in premetastatic tissues. S100A9 acts as an endogenous natural ligand for CD33//SIGLEC3 and promotes Lin‐HLA‐DR‐CD33^+^ MDSCs to secrete cellular effectors, such as IL‐10, TGF‐β, NO, and ARG molecules that inhibit hematopoiesis and promote T cell tolerance as well as myelosuppression and inflammation.^167^

##### CD39 and CD73

MDSCs also express ectonucleoside triphosphate diphosphate hydrolase 1 (ENTPD1/CD39) and 5’‐nucleotidase ecto (NT5E/CD73). CD39 catabolizes adenosine triphosphate to adenosine diphosphate/adenosine monophosphate (AMP), while CD73 mediates the subsequent catabolism of AMP to adenosine. Their expression and activity are elevated in tumor tissue and blood and are frequently associated with clinical signs of disease and poor prognosis in some cancers.^168^ The accumulation of extracellular adenosine consequently activates the differentiation of M‐MDSCs into highly immunosuppressed TAMs and reduces MHC‐I and MHC‐II expression in tumor‐infiltrating DCs,^169^ attenuating antigen‐specific T‐cell responses. Moreover, through an intracellular cascade reaction mediated by cyclic‐AMP, adenosine shifts the TME from cytotoxic T‐cell inflammation to immune tolerance, influencing the cytokines and cell morphology.^170^

##### IL‐1

IL‐1 is an inflammatory cytokine that plays a key role in tumorigenesis and progression. It is a key mediator of natural and acquired immunities and is involved in multiple pathways related to microbial recognition, activation, and functional localization of lymphocytes. IL‐1 plays different roles in tumorigenesis and progression by influencing different components of the TME.^171^ It mainly affects inflammatory processes but also has multiple immune, degradative, and progrowth properties. MDSCs secrete IL‐1 to enhance their immunosuppressive effects.^172^ Immune cells can remove senescent cells from tumors or induce cancer cell senescence by secreting proinflammatory cytokines. MDSCs have been reported to interfere with the secretory phenotype associated with tumor senescence by secreting IL‐1 receptor antagonist (IL1RN/IL‐1RA) to fight senescence in a paracrine manner. Treatments that lower IL‐1RA levels can tilt IL‐1α toward IL‐1RA homeostasis, enhancing tumor senescence and improving the efficacy of chemotherapy.^173^

IL‐1α/IL‐1 receptor signaling is located upstream of nuclear factor kappa B (NF‐κB),^174^ enhancing NF‐κB transcriptional activity and thereby regulating *PD‐1*/ *PD‐L1* expression. Malignant tumor cells suppress the immune function of T lymphocytes through pathways such as PD‐1/PD‐L1 pathway to weaken their recognition by the immune system and evade immune surveillance, achieving immune escape and prolonging the life span of tumor cells. NF‐κB also regulates TNF‐α and epidermal growth factor receptor (EGFR), forming a positive feedback regulatory loop between them to promote tumor cell proliferation.^175^

Both IL‐1α and IL‐1β promote tumor angiogenesis and invasion,^172^ but IL‐1β has a more prominent role in these processes. This difference may be attributed to the secretion of IL‐1β into the microenvironment, where it activates cells in the tumor mesenchyme, including malignant tumor cells. IL‐1β stimulates IL‐10 production by MDSCs^176^ and induces the production of proangiogenic cytokines, such as IL‐8, by endothelial cells and surrounding stromal cells.^171^

**FIGURE 5:**
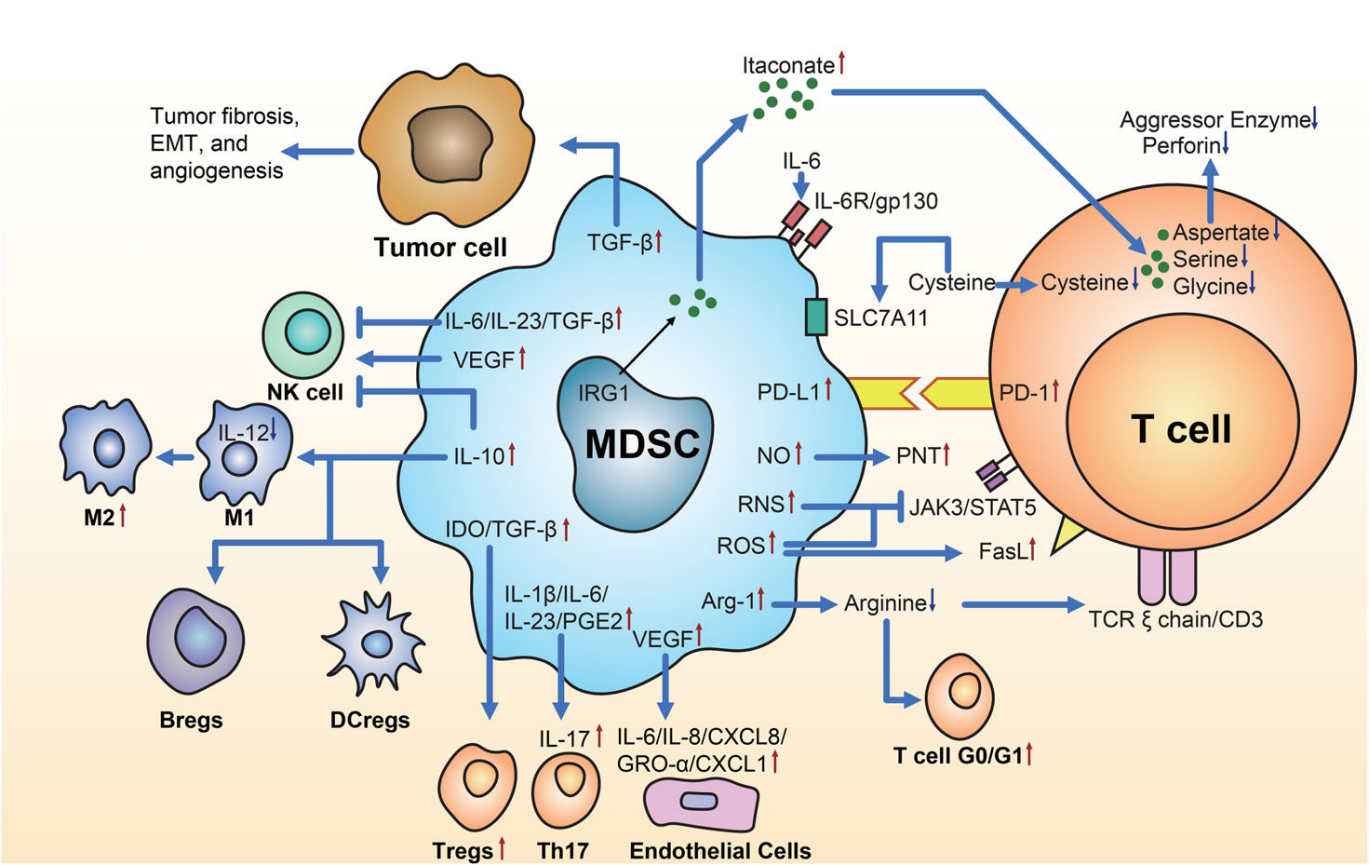
Immunosuppressive functions of myeloid‐derived suppressor cells (MDSCs). The effects of MDSCs on T cells include direct and indirect effects. The direct effects: MDSCs release ROS and RNS to act on T cells and inhibit JAK3/STAT5 signaling pathway and promote the overexpression of FasL or PD‐1 on the surface of T cells, resulting in apoptosis of T cells. MDSCs also release itaconate to repress the proliferation and antitumor cytotoxicity of T cells. The indirect effect is mainly to inhibit the development of T cells by consuming and competing for arginine and cysteine in the environment; releasing IL‐1β, IL‐6, IL‐23, PGE2, and other factors to generate Th17 and Treg cells to inhibit T cells’ immune function. At the same time, MDSCs are involved in the activation of TAMs, converting M1 to M2, mainly by increasing the release of IL‐10. VGEF may exert a proinflammatory cytokine release effect through VEGFR2/STAT3 signaling, inducing increased IL‐6, IL‐8/CXCL8, and GRO‐α/CXCL1 secretion in endothelial cells (nonleukocytes) and exerting an immunosuppressive function. VEGF upregulates the activity of NK cells and their natural immune response. In contrast, TGF‐β has involved in down‐regulating the expression of the NK‐activated receptor NKG2D on the NK cell surface, inhibiting NK cells from producing cytokines. In TME, dysregulated TGF‐β signaling suppresses antitumor immunity and promotes tumor fibrosis, epithelial‐to‐mesenchymal transition, and angiogenesis. MDSCs induce the differentiation of Bregs and DCregs through the secretion of IL‐10. In addition, IL‐10 also can inhibit the activation and proliferation of NK cells, NKT cells, and DCs and the production of various antitumor cytokines. EMT, epithelial–mesenchymal transition; Bregs, regulatory B cells; DCregs, regulatory dendritic cells; Tregs, regulatory T cells; TGF‐β, transforming growth factor‐β; VEGF, vascular endothelial growth factor; IL, interleukin; IDO, indoleamine 2,3‐dioxygenase; PGE2, prostaglandin E2; TAMs, tumor‐associated macrophages; CXCL, CXC motif chemokine ligand; GRO‐α, growth related oncogene‐α; Arg‐1, arginase‐1; ROS, reactive oxygen species; RNS, reactive nitrogen species; NO, nitric oxide; PD‐L1, programmed death ligand‐1; SLC7A11, cystine/glutamate antiporter solute carrier family 7 member 11; PD‐1, programmed death‐1; PNT, peroxynitrite; JAK3/STAT5, Janus kinase 3/signal transducer and activator of transcription 5; TCR, T‐cell receptor.

Altogether, MDSCs in the TME can induce immunosuppressive functions in various ways: affecting T cells’ generation, differentiation, migration, proliferation, and viability; inducing M2 polarization of macrophages; mediating the status of NK cells and DCs; recruiting other immunosuppressive cells; inhibiting self‐differentiation; and promoting tumor cells’ progression, EMT, and metastasis of tumor cells to construct a tumor‐promoting inflammatory environment (Figure 5).

## 5 MDSC‐TARGETED THERAPY

**FIGURE 6:**
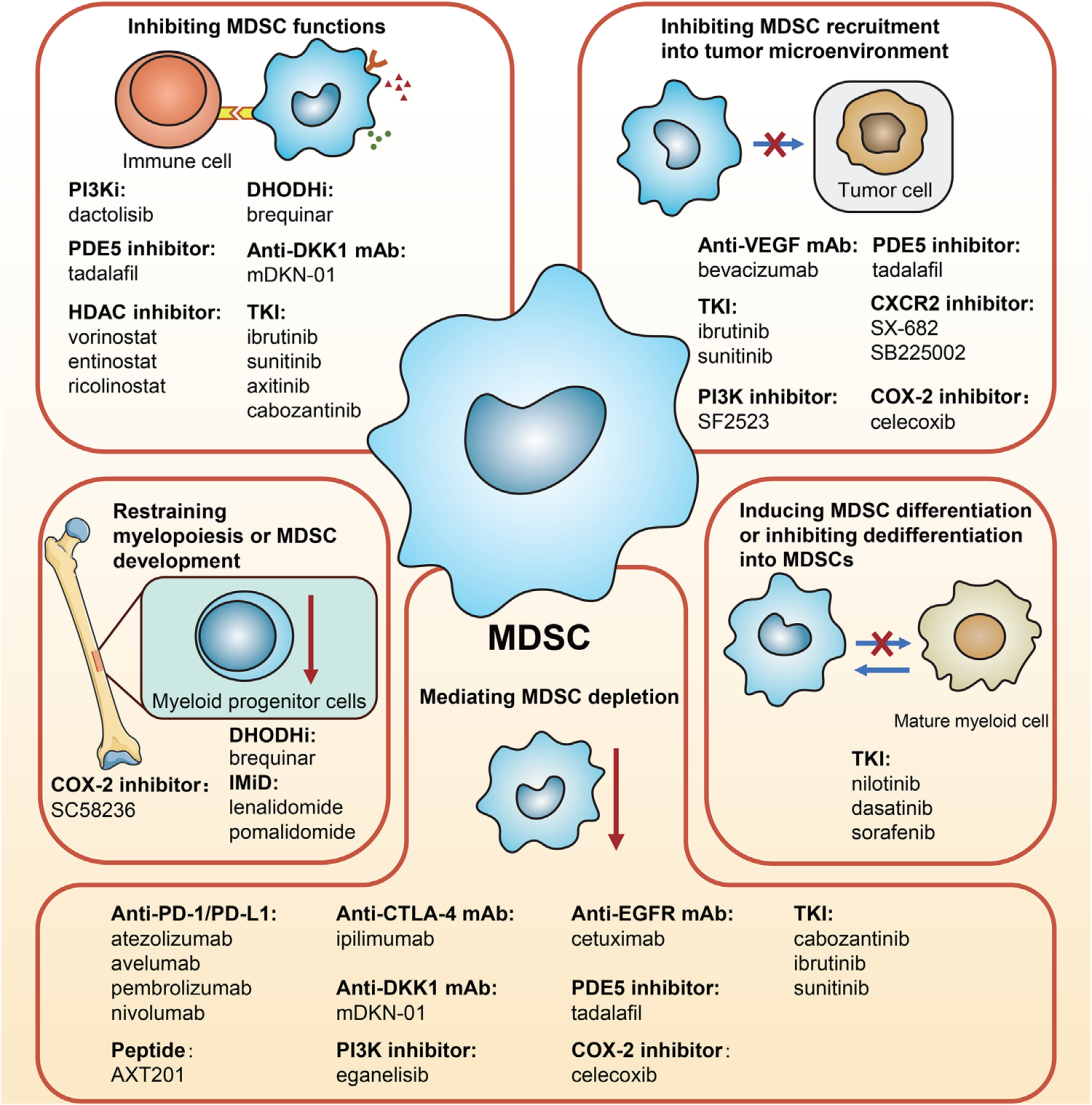
Mechanisms of myeloid‐derived suppressor cell (MDSC)‐targeted drugs in cancer therapy. MDSC‐targeted drugs can achieve anti‐MDSC effects in 5 ways: (1) inhibiting MDSC functions, (2) inhibiting MDSC recruitment into TME, (3) restraining myelopoiesis or MDSC development, (4) inducing MDSC differentiation or inhibiting dedifferentiation into MDSCs, and (5) mediating MDSC depletion. PI3K, phosphoinositide‐3‐kinase; DHODH, dihydroorotate dehydrogenase; PDE5, phosphodiesterase 5; DKK1, dickkopf‐1; HDAC, histone deacetylase; TKI, tyrosine kinase inhibitor; VEGF, vascular endothelial growth factor; CXCR2, CXC motif chemokine receptor 2; COX‐2, cyclooxygenase‐2; IMiD, immunomodulatory drug; PD‐1, programmed death‐1; CTLA‐4, cytotoxic T‐lymphocyte antigen 4; EGFR, epidermal growth factor receptor.

MDSC‐targeted drugs in cancer therapies can be divided into mAbs, small‐molecular inhibitors, and peptides based on their chemical structure. In this section, we summarize studies of MDSC‐targeted drugs and introduce their applications and mechanisms. These targeted drugs can achieve anti‐MDSC effects by inhibiting MDSC functions, inhibiting MDSC recruitment into the TME, restraining myelopoiesis or MDSC development, inducing MDSC differentiation, inhibiting dedifferentiation into MDSCs, and mediating MDSC depletion (Figure 6).

### 5.1 The applications of mAbs in MDSC‐targeted therapy

#### 5.1.1 Anti‐PD‐1/PD‐L1

PD‐1 is a self‐protective protein receptor expressed on the surface of immune cells (mainly T cell). PD‐L1 is its corresponding ligand on the surface of multiple cells, including hematopoietic and tumor cells.^177^ Receptor–ligand bonding can activate the T‐cell receptor and CD28 signaling pathways in T cells restraining of T cell proliferation, cytokine production, and immunity promotion; eventually, programmed T‐cell death occurs.^178^ Some tumor cells overexpress PD‐L1 to achieve immune escape; therefore, PD‐1 and its ligand are important targets for antitumor therapy. PD‐L1 is also expressed on the surface of MDSCs and is upregulated in the TME due to hypoxia.^179^ Therefore, anti‐PD‐1/PD‐L1 mAbs can directly act on the corresponding target in MDSCs to induce tumor suppression. The blockade of PD‐L1 in MDSCs decreases the expression of immunosuppressive cytokines, such as IL‐10, and activates T cells to resist MDSC‐induced immune suppression.^179^

In the study conducted by Tzeng et al.,^180^ the mean reduction in peripheral M‐MDSC levels reached 4.70% per dose during the successive doses of anti‐PD‐L1 treatment (atezolizumab or avelumab) targeting metastatic urothelial carcinoma. In parallel, anti‐PD‐1 mAb (pembrolizumab) decreased PD‐1‐positive M‐ and immature‐MDSCs by 1.42% and 0.73% per dose, respectively.^180^ This study did not show a correlation between MDSC PD‐1/PD‐L1 expression and prognosis after mAb treatment. However, the MDSC level before anti‐PD‐L1 mAb treatment could be regarded as a promising predictor of clinical status.^181^ Similar results of MDSC frequency reduction induced by nivolumab, a type of PD‐1 blockade, were also reported.^10^

Patients suffering from various cancers (such as melanoma and NSCLC) easily showed undesired or poor clinical effects after receiving anti‐PD‐1/PD‐L1 treatment due to drug resistance.^182^, ^183^ But fortunately, depleting ARG‐1‐expressing PMN‐MDSCs could reverse the anti‐PD‐1/PD‐L1 resistance of tumors.^184^ Targeting some other MDSC‐related immune checkpoints has reduced drug resistance and enhanced the antitumor effects of PD‐1/PD‐L1 blockades.^185^ M‐MDSCs expressing galectin 9 (*LGALS9*), the ligand of hepatitis A virus cellular receptor 2 (HAVCR2/TIM3), are correlated with reduced CD8^+^ T cell secretion. Pretreatment with anti‐TIM3 could be an effective approach for reducing resistance to nivolumab, a type of PD‐1 blockade, in patients with NSCLC.^186^ Using cabozantinib to deplete ARG‐1‐expressing PMN‐MDSCs enhanced the efficacy of anti‐PD‐1 therapy.^184^ Targeting HIF‐1α could regulate PD‐L1 expression in MDSCs, and combining anti‐VEGF/VEGFR mAbs with PD‐1 could also reduce MDSCs proliferation, improving the antitumor effects.^179^, ^187^ Given multiple PD‐1/PD‐L1 blockade resistance, personalized treatment is required, and accurate judgments of patients’ prognosis and disease progression after mAb treatment are required. Specific predictors are needed to achieve this, among which MDSCs are a promising option.^188^ However, research is still at the phenomenon observation stage; a complete mechanical explanation of MDSC‐related clinical status remains to be elucidated.

#### 5.1.2 Anti‐CTLA‐4

Whether MDSCs express CTLA‐4 remains unclear.^189^ However, some studies have shown the effects of CTLA‐4 blockade on MDSCs, especially with ipilimumab. The effects on MDSC subsets differed during ipilimumab treatment in patients with melanoma.^12^ In particular, the concentration of PMN‐MDSCs decreased in all patients undergoing CTLA‐4 blockade, while the concentration of M‐MDSCs significantly changed only in clinically benefitting patients.^12^, ^190^ Additionally, the MDSC level prior to ipilimumab treatment and its changes during therapy are highly relevant to patient survival.^191^, ^192^, ^193^ CTLA‐4 blockades combined with some other targeted drugs can reduce the frequency of MDSCs, and combinational therapies can alter MDSC functions.^194^, ^195^ Anticolony stimulating factor 1 (CSF1)/CSF1 receptor (CSF1R) treatment combined with CTLA‐4 blockade caused regression in breast and colon cancers in vivo by reprogramming MDSCs into antineoplastic ones.^195^ M‐MDSCs showed a reduced immunosuppressive ability after anti‐CTLA4 therapy combined with anti‐VEGFR therapy in mice with melanoma.^196^

#### 5.1.3 Anti‐VEGF

SET domain‐containing 2 histone lysine methyltransferase (SETD2/HIF‐1) and VEGF are abundantly activated in tumor sites, enhancing angiogenesis, which also induces MDSC migration to and differentiation at tumor sites.^197^, ^198^, ^199^ VEGF can recruit MDSCs via the JAK/STAT3 pathway and VEGF–VEGFR2 interaction^21^ and enhance their ability to generate other immunosuppressive cells.^145^ In addition, MDSCs produce VEGF to facilitate angiogenesis.^199^ Studies have observed the MDSC‐targeting effects induced by bevacizumab, an anti‐VEGF mAb.^200^, ^201^ The concentration of MDSC subsets was significantly associated with disease prognosis after treatment,^202^ with CD15^+^ M‐MDSCs increasing in abundance in patients with tumor progression. Undesirable anti‐VEGF therapeutic effects may be attributed to increase GM‐CSF levels after treatment, which mediate MDSC migration and differentiation.^203^ To reverse anti‐VEGF refractoriness in tumors, Iwai et al.^204^ considered capecitabine as a promising agent since it reduced tumor drug resistance and pyrimidine nucleoside phosphorylase‐expressing MDSCs in tumor sites and peripheral blood in patients with Lewis lung carcinoma. Low‐dose capecitabine treatment with bevacizumab also had an inhibitory effect on circulating MDSCs in patients with glioblastoma.^201^

#### 5.1.4 Anti‐EGFR

While the modulating pathways between the EGFR and MDSCs are unclear, studies have observed changes in MDSCs after interfering with the EGFR.^205^, ^206^ In T helper 1 cell cytokine‐enriched environments in pancreatic cancer, EGFR‐targeted therapy induced by EGFRBi (EGFR bispecific antibodies) armed active T cells, restraining the prostaglandin‐endoperoxide synthase 2 (PTGS2/COX2)/PGE2/ARG‐1 axis and enhancing TNF‐α, IL‐12, CCL3, CCL4, CCL5, CXCL9, and CXCL10 to suppress MDSC differentiation and accumulation.^207^ This effect may be partly attributed to EGFR interference but is more likely due to cytokine interactions between active T cells and the environment. Therefore, the T cell carrier may be further removed to determine the individual impact induced by EGFR. In clinical practice, Li et al.^206^ reported that cetuximab, an EGFR‐specific mAb, significantly decreased PMN‐MDSCs only in responders among patients with head and neck squamous cell carcinoma (HNSCC); meanwhile, it can inhibit the polarization of monocytes into M2 macrophages and induce functioning by bonding Fc‐gamma receptors in myeloid cells. In nonresponders, the number of circulating M‐MDSCs significantly increased. Another clinical trial showed that a higher pretreatment M‐MDSC/PMN‐MDSC ratio might induce a higher pseudo F‐statistic in patients with HNSCC.^208^ Therefore, M‐MDSCs could be a promising predictor of clinical prognosis after cetuximab treatment. However, combinational therapy with cetuximab and radiation markedly upregulated IL‐6–STAT3 signaling in circulating MDSCs and might lead to undesirable patient prognoses.^209^

### 5.2 The applications of small‐molecular inhibitors in MDSC‐targeted therapy

#### 5.2.1 Tyrosine kinase inhibitors

Sunitinib is a type of receptor protein‐TKI (RPTKI) with multiple targets, including VEGFR, ret proto‐oncogene (RET), and KIT proto‐oncogene receptor tyrosine kinase (KIT/c‐Kit),^210^ that reduced the peripheral MDSC frequency^8^ and part of the MDSC frequency in tumor sites.^9^ Mechanistically, its inhibitory effects on MDSCs might inhibit the VEGFR/VEGF axis since it functions in the recruitment of immunosuppressive cells, such as MDSCs and Tregs.^145^ Sunitinib‐induced MDSC inhibition is related to STAT3 pathway downregulation, constraining the development of MDSCs from myeloid progenitor cells^8^ and improvement of angiogenesis in tumor sites.^211^ Similarly, directly targeting STAT3 is also an effective approach for inhibiting the generation of M‐MDSCs which has been observed in patients with prostate cancer.^212^ Interestingly, the anti‐MDSC effect of sunitinib is independent of its antitumor activities since significant effects on MDSCs have occurred in the absence of tumor progression inhibition.^13^ c‐Kit is another promising target for anti‐MDSC therapies, which accounts for MDSC accumulation via enhanced myelopoiesis and inhibited myeloid cell differentiation,^213^ and sunitinib has demonstrated a c‐kit‐dependent anti‐MDSC activity.^214^ However, sunitinib resistance also occurs in tumors due to the recombinant GM‐CSF, which selectively activates STAT5 and suppresses STAT3 instead.^13^ Additionally, GM‐CSF induces the transformation of two MDSC subsets, specifically^215^ to gain higher immunosuppressive activities in the TME.^216^, ^217^

Other TKIs have also shown anti‐MDSC efficacies. Stiff et al. showed that ibrutinib, a type of Bruton's TKI, could weaken NO and IDO production in MDSCs and MDSC cytopoiesis and migration.^218^ Another TKI, cabozantinib, significantly decreased the MDSC numbers in both the tumor site and spleen, restraining ARG‐1 expression and assisting the recruitment of CD8^+^ T cells into the TME.^219^ Unlike sunitinib, some TKIs, including nilotinib, dasatinib, and sorafenib, have shown dose‐dependent inhibitory effects on the differentiation process of mature monocytes into M‐MDSCs but not on the maturation process of M‐MDSCs as well as their function, suggesting that these inhibitors are more applicable in the early stage of MDSC infiltration.^220^ However, the MDSC‐modulating patterns of TKIs and the different regulatory pathways of sunitinib compared with other TKIs require further studies.

#### 5.2.2 CXCR inhibitors

Chemokines are important mediators in tumor progression. The CXCR–CXCL system can induce immune cell infiltration, tumor proliferation, metastasis, and angiogenesis.^221^ CXCL1, CXCL2, CXCL5, and CXCL8 can mediate MDSC recruitment and accumulation at the tumor site via CXCL–CXCR1/CXCR2 interactions.^222^ SX‐682, a selective CXCR1/CXCR2 inhibitor, has shown antitumor capability via MDSCs: suppressing MDSC recruitment in the TME.^223^, ^224^ CXCR2 inhibition suppressed the metastasis of pancreatic ductal adenocarcinoma^225^ and enhanced the efficacy of immune checkpoint inhibitors (ICIs) by increasing T cell infiltration.^225^, ^226^ However, Sun et al.^224^ indicated that the antitumor activity of SX‐682 is separate from its effect on MDSCs, partly because it only inhibits the migration of PMN‐MDSCs merely without down‐regulating their function and tumor CXCL expression. Therefore, combining of CXCR blockades with ICIs is essential. CXCR2 is also a mediator of MDSC recruitment in regional lymph nodes, playing a role in preventing tumor metastasis, and CXCR‐antagonist SB225002 reversed MDSC trafficking.^227^ Moreover, the CXCL12/CXCR4, CXCL13/CXCR3, and CXCL17/CXCR8 axes also induce MDSC migration to tumor sites.^222^ CXCR4 inhibitors potentiated anti‐PD‐1 ability in pancreatic cancer, ovarian cancer, and glioblastoma.^228^, ^229^, ^230^ In a phase IIa clinical trial, combining the CXCR4 inhibitor BL‐8040 with the PD‐1 mAb pembrolizumab decreased the PMN‐MDSC density in patients with pancreatic ductal adenocarcinoma; one stable patient showed reduced PMN‐MDSC levels (by approximately 25%) after two cycles of combined treatment.^229^ No published studies support the inhibition of the CXCL13/CXCR3 and CXCL17/CXCR8 axes.

#### 5.2.3 Immunomodulatory drugs

Immunomodulatory drugs (IMiDs), consisting of lenalidomide and pomalidomide, are a group of antitumor drugs derived from the prototype compound thalidomide that can be used for some hematologic tumors, especially multiple myeloma.^231^, ^232^ Lenalidomide reverses tumor progression mainly via cytotoxicity and angiogenesis resistance,^233^ with its antitumor mechanisms attributable to MDSC inhibition.^234^, ^235^ Sakamaki et al.^233^ found a significant 1.39% reduction in splenic MDSCs in tumor‐bearing but not in naïve mice. Since lenalidomide is a cereblon‐targeting (CRBN‐targeting) drug, comparing *CRBN* expression in MDSCs from naïve and tumor‐bearing individuals is necessary. Kuwahara‐Ota et al.^236^ found that combining lenalidomide with pomalidomide, inhibited MDSCs by downregulating CCL5 and macrophage migration inhibitory factor in myeloma cells, enhancing *IRF8* expression and downregulating CCR5 in peripheral blood mononuclear cells in vitro. Notably, the multiregulatory mechanism of lenalidomide could also increase CD14^+^CD15^+^ MDSCs, causing T cell suppressive effects.^237^ Therefore, it is worth exploring the optimal concentration that provides good antitumor efficacy with minimal immunosuppressive activity.

#### 5.2.4 IDO inhibitors

Tryptophan is an essential amino acid required that is taken in through diet, and tumor burden is associated with tryptophan catabolism. Initially, this kind of catabolism, which results in the depletion of tryptophan, is controlled by IDO and tryptophan dioxygenase. Tryptophan starvation suppresses T cell growth and activation, promoting tumor immune escape, metastasis, and angiogenesis.^238^, ^239^ Physiologically and initially, IDO1, which induces the establishment of self‐tolerance in the fetal period, facilitates immunosuppressive behaviors by promoting the generation of MDSCs and forkhead box P3 (FOXP3)^+^ Tregs.^240^, ^241^ In the TME, tumor IDO promotes MDSC recruitment, infiltration, and activation via Treg‐dependent pathways.^242^ Consequently, MDSCs in breast cancer stimulated with the IL‐6 family showed increased *IDO1* expression and assisted in tryptophan catabolism partly via the STAT3/NF‐κB/IDO pathway, which forms positive feedback.^243^ Epacadostat is a selective IDO1 inhibitor, which has antineoplastic effects via facilitating T and NK cell proliferation and immune activity, increasing the number of IFN‐γ and CD86^hi^ DCs, and reversing the accumulation of Treg‐like cells.^244^ A phase II clinical trial reported that the mean MDSC level decreased by 1.9% from the baseline after epacadostat treatment.^245^ Navoximod and indoximod are other IDO1 inhibitors with tumor growth suppressive activity but generally lack information about MDSC‐related changes. Only a few studies have reported the correlation between IDO1 inhibitors and MDSCs; thus, further research is needed to bridge this gap.

#### 5.2.5 Phosphoinositide‐3‐kinase inhibitors

Once activated by upstream signals from receptor tyrosine kinases, phosphoinositide‐3‐kinases (PI3Ks) will lead to several key regulations in tumor‐associated immunity.^246^ The PI3K signaling pathway has diverse protumoral abilities: promoting the production of immunosuppressive cytokines and enhancing *PD‐L1* expression and MDSC and Treg infiltration.^247^ Class I PI3K has four isoforms, namely α, β, δ, and γ. However, anti‐MDSC activity is only observed with PI3Kγ inhibitors (IPI‐549) both in vivo and in vitro.^248^, ^249^ In the in vivo murine study conducted by Zhang et al.,^248^ IPI‐549 decreased CD11b^+^ Gr‐1^+^ MDSCs in pancreatic cancer by almost 50% via two distinct delivery routes: oral administration and a targeted nanoparticle‐encapsulated approach. Nanoparticle usage doubled the half‐life of IPI‐549 and ensured drug accumulation in the tumor site, but the MDSC concentration did not significantly differ between the two distinct routes, suggesting that the anti‐MDSC effects of IPI‐549 may be dose‐independent. Additionally, the dual PI3K/mechanistic target of rapamycin kinase (mTOR) inhibitor dactolisib downregulated the oxidative phosphorylation of MDSCs.^250^ The dual PI3K/mTOR inhibitor SF2523 has also been reported to block CD11b^+^ Gr‐1^+^ MDSC recruitment at tumor sites.^251^

#### 5.2.6 Phosphodiesterase 5 inhibitors

Phosphodiesterase (PDEs) are common metallohydrolases downstream of cyclic‐AMP and cyclic‐GMP that induce the inactivation of both secondary messengers. Among the 11 subtypes of PDEs, PDE5 has a cGMP‐specific hydrolytic function.^252^ PDE5 inhibitors have been developed and used for various cancer types, including HNSCC, melanoma, myeloma, prostate cancer, gastrointestinal cancer, and neurologic tumors,^253^, ^254^, ^255^, ^256^, ^257^ augmenting antitumor immune responses in cancer‐bearing hosts. The targeted roles of PDE5 inhibitors are observed in MDSCs by competently suppressing the function and recruitment of MDSCs and decreasing the concentration of MDSCs via two pivotal pathways: reduction of ARG‐1 and iNOS production, which induces T‐cell dysfunction.^258^ *ARG‐1* and *iNOS* expressions are mediated by STAT6/IL‐4 receptor alpha (IL‐4Rα), and PDE5 inhibitors can affect outcomes by reducing of IL‐4 Rα in the upstream pathway.^258^, ^259^ Moreover, the decrease in ROS induced by PDE5 inhibitors also benefits to tumor‐specific immunity restoration.^256^ In both murine tumor models and clinical trials, tadalafil has been shown to have potential utility in MDSC‐targeted treatments. Noonan et al.^256^ found that the number of CD14^+^ MDSCs decreased by 5.6% in patients with end‐stage multiple myeloma after 11 months of treatment with tadalafil. Given its influence on MDSC function, IL‐4Rα expression markedly decreased from 51.37 to 2.13% after treatment, and both IL‐4Rα and ROS levels were restored to baseline.^256^ Similarly, a phase II clinical trial conducted on patients with HNSCC suggested that MDSC generation and function were significantly impaired after tadalafil therapy.^260^ Notably, a high PDE5 inhibitor intake may result in contrasting effects on antitumor immunity modulation since they may cause off‐target effects on PDE11 at high doses, which mediates the increase in cyclic‐AMP.^261^

#### 5.2.7 Histone deacetylase inhibitors

Vorinostat, entinostat, and ricolinostat are histone deacetylase (HDAC) inhibitors that promote histone acetylation, transforming gene expressions via epigenetic reprogramming.^262^ HDAC inhibitors are proven to have shown inhibitory functions in MDSCs of breast, renal, and lung cancers; neuroblastoma; and lymphoma.^262^, ^263^, ^264^, ^265^ The principal effect of entinostat is to inhibit the functions of PMN‐MDSCs, but its effect against M‐MDSCs remains unclear.^263^, ^266^ Entinostat can reduce ARG‐1, iNOS, and COX‐2 production and modulate NF‐κB‐ and STAT3‐related pathways.^264^, ^266^ The side effect is that entinostat alone may increase the number of PMN‐MDSCs,^265^, ^266^ therefore, combined treatment with other targeted drugs can make full use of MDSC inhibitory functions. Combined with 5‐azacytidine and DNA methyltransferase inhibitors, entinostat restrained the trafficking of MDSCs and promoted their differentiation.^267^ In addition, entinostat‐ICI combinations altered the TME from an M‐MDSC‐ to PMN‐MDSC‐predominant milieu and improved tumor sensitivity to ICIs.^265^, ^266^

#### 5.2.8 COX‐2 inhibitors

PGE2 is an inflammatory cytokine that tumors secrete to create a local proinflammatory microenvironment.^268^, ^269^, ^270^ COX‐2 is the enzyme that induces PGE2 production. Its expression in the TME can promote tumor proliferation and angiogenesis and reduce tumor apoptotic and immunologic responses.^270^ PGE2 can induce MDSC maturation from bone marrow stem cells^271^ and promote MDSC accumulation via COX2/PGE2/prostaglandin E receptor 4 (PTGER4/EP4) signaling.^272^ The COX2 inhibitor celecoxib reduced the number and immunosuppressive ability of MDSCs by reducing ROS and NO production and reversing MDSC‐related T‐cell tolerance.^273^, ^274^ Furthermore, GM‐CSF production is suppressed, which affects MDSC maturation and accumulation.^275^ Another COX‐2 inhibitor, SC58236, inhibited MDSC differentiation in breast cancer.^271^

#### 5.2.9 Dihydroorotate dehydrogenase inhibitor

Colligan et al.^276^ recently found that the dihydroorotate dehydrogenase (DHODH) inhibitor brequinar, once used to treat leukemia by restoring terminal differentiation of leukemic myeloid progenitors, could successfully target MDSCs in a breast cancer model. DHODH accelerates pyrimidine synthesis,^277^ an important step leading to MDSC biogenesis and immunosuppression. DHODH inhibition ultimately promotes the terminal differentiation and maturation of myeloid cells, which is already achieved in vitro in human bone marrow, and the antitumor immunity of T cells.^276^ Combining brequinar with an anti‐PD‐1 mAb overcame the drug resistance of breast cancer and restored CD8^+^ T cell proliferation and CD8^+^ T cell‐mediated direct tumoricidal effects.^276^

### 5.3 The applications of peptides in MDSC‐targeted therapy

Tumor‐targeting peptide is a state‐of‐the‐art research direction for tumor‐related targeted treatments. Peptides are notable for their small molecule mass, synthetic convenience, and good biocompatibility compared with proteins. The peptides used to target MDSCs, or broadly the TME can be divided into two categories: (1) those without antitumor efficacies used only in targeted combinations of specific receptors, and (2) those with specific antitumor functions. The first group of peptides functions as carriers of antitumor drugs. The pharmacological actions of antitumor drugs can be achieved via specific binding to superficial, intracellular, or intranuclear receptors. Most studies have focused on delivering drugs to tumor cell surfaces or targeting vascular endothelial cells to block tumor nutrient sources. FAM‐A54, selected by phage display approaches, has shown binding specificity for diverse liver cancer cell lines with the potential binding motif of Pro‐Ser. A54‐covered doxorubicin has shown a high absorptivity in cancer cells.^278^ The tripeptide NGR (Asn‐Gly‐Arg) was notably competent for anchoring tumor‐related angiogenic vessels, molecularly targeting aminopeptidase N (ANPEP/CD13) in endothelial cells.^279^ Zheng et al.^280^ synthesized an NGR‐apoprotein fusion protein and loaded active enediyne chromophore to realize lidamycin carrying. Lidamycin comprises two components, apoprotein and the active enediyne chromophore, and is considered a common chemotherapeutic drug that damages DNA. Tumor growth inhibition was reportedly elevated by 14.1% compared with tumor suppression after a single lidamycin treatment. The antitumor effects of doxorubicin can also be enhanced by NGR.^281^ While minimal evidence has shown that NGR alone can affect MDSCs, the NGR–TNF complex (low dose: 0.8 μg/m^2^) does not induce MDSC mobilization in peripheral blood and recruitment to the tumor site.^282^ Therefore, NGR–TNF antitumor treatment excludes the drug‐fast impact of MDSCs. RGD (Arg‐Gly‐Asp) is another tripeptide specifically recognizing integrin αvβ3, Xia et al.^283^ synthesized a RGD‐contained, lactate dehydrogenase A (LDHA) small inhibitory RNA‐cored nanostructure assembly, this specific assembly could inhibit LDHA expression, which subsequently decreased G‐CSF and GM‐CSF production and suppressed MDSC recruitment. Another peptide AXT201, a 20‐amino polypeptide, achieves antitumor effect by the reduction of protumor growth factors, such as VEGF, HGF, and insulin‐like growth factor 1, inhibition of angiogenesis, remodeling of the TME, and promotion of CD8^+^ T cell activation.^284^ AXT201 was shown to mainly alter the proportion of MDSC subtypes in triple‐negative breast cancer to realize an antitumor change in the TME. In particular, M‐MDSCs decreased by 28%, close to sunitinib's effects on the entire MDSC population by sunitinib (33%).^13^ However, AXT201 had minimal impact on PMN‐MDSCs.^284^ The mechanisms underlying M‐MDSC reduction mechanisms may be attributed to the coreceptor and the corresponding pathways of AXT201 since MDSCs can promote tumor angiogenesis by producing VEGF, MMP9, and bFGF.^199^, ^285^ Anti‐VEGF treatment also significantly affects MDSCs as mentioned above. While this biomimetic peptide might also modulate other growth factors, further research is required. MDSC‐targeted drugs and studies reporting their effectiveness are summarized in Table 2.

**TABLE 2:** Drugs and studies to target myeloid‐derived suppressor cells (MDSCs) in cancers.

Drug type	Drug name	Year	Cancer samples	Effects	Drug status	References
Monoclonal antibody	Atezolizumab/avelumab (anti‐PD‐L1 mAb) + pembrolizumab (anti‐PD‐1 mAb)	2018	Metastatic urothelial carcinoma blood samples (human)	The percentage of PDL1+ monocytic myeloid‐derived suppressor cells (M‐MDSC) decreased after anti‐PDL1 treatment. The percentage of PD1+ M‐MDSC and immature‐myeloid‐derived suppressor cells (I‐MDSC) decreased after anti‐PD1 treatment.	Preclinical trial	^180^
	Atezolizumab (anti‐PD‐L1 mAb)	2018	Non‐small cell lung cancer (NSCLC) blood samples (human)	MDSC frequency was decreased .	Preclinical trial	^11^
	RMP1–14 (anti‐PD‐1 mAb) + peptide R (CXCR4 antagonist)	2019	MC38 colon cancer cell line (mice); B16 melanoma transduced with human CXCR4 cell line (mice)	Increase the CD8/MDSC ratio	Preclinical trial	^286^
	Nivolumab (anti‐PD‐1 mAb) + temozolomide (alkylating agents)	2020	Small cell lung cancer (SCLC) blood samples (human)	MDSC experienced an early decrease during therapy	Phase II clinical trial NCT03728361	^10^
	Ipilimumab (anti‐ CTLA‐4 mAb) + ATRA (all‐trans retinoic acid)	2018	Melanoma blood samples (human)	ATRA reduces PD‐L1, IL‐10, and IDO gene expression by MDSCs. ATRA‐ipilimumab combination significantly decreases the frequency of circulating MDSCs.	Phase II clinical trial NCT02403778	^287^
	Cetuximab (anti‐EGFR mAb)	2015	Locally advanced head and neck squamous cell carcinoma (HNSCC) blood samples (human)	The M‐MDSCs of nonresponders increased and the polymorphonuclear myeloid‐derived suppressor cells (PMN‐MDSCs) of responders decreased significantly.	Phase II clinical trial NCT01218048	^206^
	Bevacizumab (anti‐VEGF mAb)	2016	Non‐small cell lung cancer (NSCLC) blood samples (human)	The decrease of PMN‐MDSCs and the increase of CD15^+^ M‐MDSCs were shown in patients with disease progression.	Preclinical trial	^202^
	Bevacizumab (anti‐VEGF mAb) + EGFR TKI	2018	EGFR‐mutated lung adenocarcinoma blood samples (human)	The percentage of circulating MDSCs decreased after bevacizumab–TKI combined treatment while TKI alone had no significant changes.	Phase II clinical trial	^200^
	N‐809 (IL‐15 superagonist N‐803 with two αPD‐L1 domains)	2020	4T1 triple negative breast carcinoma cell line (mice) Yac‐1 lymphoma cell line (mice) MC38 colon carcinoma cell line (mice)	Decrease the M‐MDSC infiltration in primary tumor tissues, but increase the concentration of M‐MDSCs in the draining lymph nodes.	Preclinical trial	^288^
	MR16–1 (anti‐IL‐6R mAb)	2012	CMC‐1 methylcholanthrene‐induced skin squamous cell carcinoma cell line (mice)	Anti‐IL‐6R mAb reduced the number of MDSCs, subsequently improving antitumor T cell response and IFN‐γ expression.	Preclinical trial	^289^
	mDKN‐01 (anti‐DKK1 mAb)	2021	B16F0 melanoma cell line (mice); 4T1 breast cancer cell line (mice)	Reduction of Gr‐1+CD11b+ MDSCs in the tumor and spleen Upregulation of PD‐L1 expression on MDSCs	Preclinical trial	^290^
Small‐molecule inhibitor	Ibrutinib (inhibitor of Bruton's tyrosine kinase and IL2‐inducible T‐cell kinase)	2016	4T1 breast cancer cell line (mice) B16F10 melanoma cell line (mice) metastatic melanoma blood samples (human)	Ibrutinib reduced the NO production, MDSC migration, and the frequency of MDSCs with significance. It enhanced the antitumor effect of PD‐L1 blockade as well.	Preclinical trial	^218^
	Ibrutinib (inhibitor of Bruton's tyrosine kinase and IL2‐inducible T‐cell kinase)	2021	NB9464 neuroblastoma cell line (mice)	The treatment of ibrutinib on MDSCs changed the production of NO and reduced *Ido*, *Arg* and *Tgfβ* mRNA expression.	Preclinical trial	^291^
	Ibrutinib (inhibitor of Bruton's tyrosine kinase and IL2‐inducible T‐cell kinase)	2021	Chronic lymphocytic leukemia blood samples (human)	The number of PMN‐MDSCs, not M‐MDSCs, decreased consistently after treatment, and the decrease in PMN‐MDSC number was related to CLL level.	Preclinical trial	^292^
	Eganelisib (IPI‐549) (Phosphoinositide‐3‐kinases inhibitor)	2019	KPC pancreatic cancer cell line (mice)	The decrease of MDSCs was shown after IPI‐549 oral treatment or polymeric nanoparticle treatment.	Preclinical trial	^248^
	SX‐682 (CXCR2 inhibitor)	2020	MOC2 oral cancer (mice) Head and neck squamous cell carcinoma (HNSCC) tumor biospecimens and blood samples (human)	SX‐682 can significantly decrease the trafficking of MDSCs into TME, thus causing the disinhibition of NK cells.	Preclinical trial	^223^
	SX‐682 (CXCR2 inhibitor)	2019	MOC1 oral cancer cell line (mice) LLC Lewis lung carcinoma cell line (mice)	SX‐682 induced the decrease of CXCR2‐positive PMN‐MDSCs without altering CXCR2 ligand expression.	Preclinical trial	^224^
	Lenalidomide (CC‐5013) (ligand of ubiquitin E3 ligase cereblon)	2014	A20 lymphoma cell line (mice)	Lenalidomide reduced the splenic MDSCs without reducing tumor burden.	Preclinical trial	^233^
	Lenalidomide (CC‐5013) (ligand of ubiquitin E3 ligase cereblon)	2018	MM1.s multiple myeloma cell line (human) Multiple myeloma blood samples (human)	The lenalidomide‐based treatment reduced both the number of PMN‐MDSCs and ARG‐1 expression.	Preclinical trial	^234^
	Tadalafil (PDE 5 inhibitor)	2014	Multiple myeloma tumor biospecimens and blood samples (human)	Tadalafil reduced the frequency of M‐MDSCs as well as ARG‐1, iNOS, and ROS production of MDSCs. Changes were more significant in tumor tissues than periphery.	Preclinical trial	^256^
	Sunitinib (receptor TKI)	2009	786‐O and RCC4 renal cell carcinoma (RCC) cell lines (human) RENCA renal carcinoma cell line (mice)	Sunitinib suppressed Stat3 expression in renal cell carcinoma‐related MDSCs.	Preclinical trial	^293^
	Sunitinib (receptor TKI)	2009	MCA26 colon carcinoma cell line (mice) LLC1 Lewis lung carcinoma cell line (mice)	Sunitinib reduced the frequency of MDSCs, suppressive activity of MDSCs as well as the PD‐L1 expression on MDSCs.	Preclinical trial	^214^
	Sunitinib (receptor TKI)	2009	renal cell carcinoma (RCC) blood samples (human)	Sunitinib reduced MDSC viability and function significantly, which were associated with the reversal of type 1 T‐cell inhibition and CD3^+^CD4^+^CD25^hi^Foxp3^+^ Treg activation.	Preclinical trial	^294^
	Sunitinib (receptor TKI)	2010	4T1 mammary carcinoma (mice) CT26 colonic carcinoma (mice) RENCA renal carcinoma (mice)	Both M‐MDSCs and PMN‐MDSCs were suppressed by sunitinib, and MDSC resistance was induced by GM‐CSFs generation.	Preclinical trial	^13^
	Sunitinib (receptor TKI)	2011	Metastatic renal cell carcinoma (mRCC) blood samples (human) 4T1 mammary carcinoma cell line (mice) CT26 colonic carcinoma cell line (mice) RENCA renal carcinoma cell line (mice)	Sunitinib reduced the tumor‐infiltrating MDSCs in RENCA and CT26, but not 4T1, bearing mice. Sunitinib can also reduce circulating MDSCs accumulation in mRCC patients significantly, but MDSC resistance may happen.	Preclinical trial	^9^
	Sunitinib (receptor TKI)	2015	Blood samples of various cancer patients with oligometastases (human)	Sunitinib decreased the number of M‐MDSCs as well as Arg levels in M‐MDSCs.	Preclinical trial	^295^
	Sunitinib	2015	Renal cell carcinoma (RCC) tumor biospecimens (human)	The sunitinib‐related MDSC suppression improved tumor‐infiltrating lymphocyte expansion.	Preclinical trial	^296^
	Sunitinib (receptor TKI)	2019	Nonmuscle invasive bladder cancer (NMIBC) blood samples (human)	CD33^+^ MDSCs decreased significantly after cycle 2 and 3 of the sunitinib treatment.	Phase II clinical trial NCT01118351	^297^
	Sunitinib (receptor TKI)	2019	TC‐1 cell line (mice)	Both MDSCs in tumor site and spleen decreased after treatment.	Preclinical trial	^8^
	Nilotinib, dasatinib, sorafenib, and sunitinib (TKIs, TKIs)	2016	LX2 hepatic stellate cell line (human)	Nilotinib, dasatinib, and sorafenib, but not sunitinib decreased the differentiation of monocytes into M‐MDSCs. both the MAPK and NF‐κB pathways were not affected by the applied TKIs.	Preclinical trial	^220^
	INCB024360 (indoleamine 2,3‐dioxygenase inhibitor, IDO inhibitor)	2018	Myelodysplastic syndromes bone marrow samples (human)	Most patients experienced a decline on bone marrow MDSCs.	Phase II clinical trial NCT01822691	^245^
	Vorinostat (histone deacetylase inhibitor, HDAC inhibitor)	2016	9464D neuroblastoma cell line (mice)	Vorinostat decreased the number and function of MDSCs.	Preclinical trial	^262^
	Entinostat (histone deacetylase inhibitor, HDAC inhibitor)	2017	RENCA‐Luc renal cell carcinoma cell line (mice) LLC lung carcinoma cell line (mice) CT26 colon carcinoma cell line (mice)	Entinostat suppressed MDSC functions by reducing ARG‐1, iNOS, and COX‐2 production of MDSCs.	Preclinical trial	^264^
	Entinostat (histone deacetylase inhibitor, HDAC inhibitor) + immune‐checkpoint inhibitors (ICI)	2018	NT2.5 mammary cancer cell line (mice)	The Entinostat‐ICI combined therapy altered the TME from M‐MDSC‐ to PMN‐MDSC‐predominant milieu, and reduced ARG‐1 production of MDSCs.	Preclinical trial	^265^
	Entinostat (histone deacetylase inhibitor, HDAC inhibitor) Ricolinostat (histone deacetylase inhibitor, HDAC inhibitor)	2020	EL4 lymphoma cell line (mice) LLC lung carcinoma cell line (mice)	Entinostat can only inhibit the immunosuppressive functions of PMN‐MDSCs, while ricolinostat can inhibit the functions of M‐MDSCs, the combination of two HDACis is capable of targeting two major MDSC subsets.	Preclinical trial	^263^
	Entinostat (histone deacetylase inhibitor, HDAC inhibitor) + 5‐azacytidine + DNA methyltransferase	2020	LLC1 Lewis lung carcinoma cell line (mice) 4T1 mammary cancer cell line (mice) HNM007 p53‐null esophageal squamous cell carcinoma cell line (mice)	The combined therapy downregulated CCR2 and CXCR2 to inhibit the trafficking of MDSCs, and promoted MDSC differentiation into a more‐interstitial macrophage‐like phenotype.	Preclinical trial	^267^
	Axitinib (TKI of VEGFR‐1, ‐2, and ‐3)	2015	MO4 melanoma cell line (mice)	Splenic and tumor‐infiltrating M‐MDSCs had a decline on immunosuppressive capacity after treatment. And axitinib‐treated MDSCs can stimulate allogeneic T cells, differentiating toward an antigen‐presenting phenotype.	Preclinical trial	^298^
	Celecoxib (cyclooxygenase‐2 inhibitor, COX‐2 inhibitor)	2010	AB1 mesothelioma cell line (mice)	Celecoxib reduced the number of MDSCs, reduced ROS and NO production and reversed T cell tolerance of MDSCs.	Preclinical trial	^274^
	Celecoxib (cyclooxygenase‐2 inhibitor, COX‐2 inhibitor) Sunitinib (receptor TKI)	2017	RENCA renal carcinoma cell line (mice) 769‐p renal carcinoma cell line (human)	GM‐CSF expression can be inhibited by both celecoxib and sunitinib. The combined therapy reduced the number of circulating MDSCs as well as STAT3 expression in MDSCs.	Preclinical trial	^275^
	SC58236 (cyclooxygenase‐2 inhibitor, COX‐2 inhibitor)	2007	4T1 mammary cancer (mice)	SC58236 retarded MDSC development from bone marrow stem cells.	Preclinical trial	^271^
	Brequinar (dihydroorotate dehydrogenase inhibitor, DHODH inhibitor)	2022	4T1 breast cancer cell line (mice); bone marrow culture system (human)	Brequinar retrained pyrimidine synthesis in myeloid cells in human bone marrow to inhibit MDSC biogenesis and suppress the function of MDSCs, enhancing T cell immunity. The combination of brequinar and anti‐PD‐1 can relieve the ICI resistance of breast cancer and promote CD8^+^T‐induced cytotoxicity against tumor cells.	Preclinical trial	^276^
	Cabozantinib (TKI) + Anti‐HER2 mAb	2022	4T1 breast cancer cell line (mice)	The number of MDSCs in TME, lymph nodes, and spleens decreased by 20, 0.8, and 35% after the combination of cabozantinib and anti‐HER2 mAb. the combined treatment also inhibited the Arg 1 expression on MDSCs and recruited CD8^+^T cells into tumor site, tumor‐draining lymph nodes, and splenic lymphocytes	Preclinical trial	^219^
	Dactolisib (Phosphoinositide‐3‐kinases inhibitor, PI3K inhibitor) + anti‐CTLA4 mAb + anti‐PD1 mAb	2017	Prostate cancer (mice)	Dactolisib suppressed the PI3K signaling of MDSCs, upregulated interleukin‐1 receptor antagonist, and retain the activity of CD4^+^ and CD8^+^ T cells, finally promoting the antitumor effect of anti CTLA4 and anti‐PD1 mAbs.	Preclinical trial	^299^
	Dactolisib (Phosphoinositide‐3‐kinases inhibitor, PI3K inhibitor)	2020	Prostate cancer (mice)	Dactolisib inhibited mTOR and oxidative phosphorylation on PMN‐MDSCs, and therefore downregulate fatty acid oxidation and cause MDSC immunosuppressive activity.	Preclinical trial	^250^
Peptides	AXT201 (an integrin‐binding peptide)	2020	Triple‐negative breast cancer cell line (mice)	M‐MDSC showed a 28% reduction after AXT201 treatment, while PMN‐MDSC did not demonstrate significant change.	Preclinical trial	^284^
	R‐mPDV/PDV/DOX/siL (redox‐responsive nanoassembly)	2021	4T1 Breast cancer cell line (mice)	R‐mPDV/PDV/DOX/siL induced LDHA silencing, suppressing the production of G‐CSF and GM‐CSF and leading to the inhibition of MDSCs recruitment.	Preclinical trial	^283^

### 5.4 Potential MDSC‐targeted therapeutic methods dependent on ferroptosis inhibition

Previous studies have revealed that drug‐resistant tumors are especially sensitive to ferroptosis, indicating the promising effect of ferroptosis in tumor therapy.^300^, ^301^ On the contrary, ferroptosis also release damage‐associated molecular patterns to disturb antitumor innate immunity.^302^ Meanwhile, induction of ferroptosis can cause bone marrow injury as the main obstacle for antitumor usage of ferroptosis activators.^303^ Therefore, the clinical application of ferroptosis activators still has left many problems to fix. As mentioned above, taking TME into consideration, ferroptosis promotes the immunosuppressive activities of PMN‐MDSCs. Specifically inhibiting the ferroptosis of MDSCs may enhance antitumor immunity and consolidate the efficacy of other antitumor drugs, and research has already revealed that inhibiting ROS, the critical effector of ferroptosis, in MDSCs by catalase can induce T cell rejuvenation.^304^ So far, there are no treatments proposed to specifically inhibit ferroptosis of MDSC in tumor‐bearing patients, in the future, MDSC characteristic receptor binding protein (such as targeted peptides) and ferroptosis inhibitors can be combined to become an alternative to accurately target MDSC through drug carriers.

## 6 CONCLUSIONS

MDSC is a kind of immunosuppressive cell which plays a vital role in various inflammatory diseases and tumor pathogenesis, progression as well as metastasis. Several studies have described the characteristics of MDSCs, dividing them into subtypes and elaborating on their functions in pathological conditions. Under most inflammatory circumstances, their immunosuppressive capacity can worsen inflammation progression. But in some scenarios such as hyperinflammation and autoimmune diseases, inhibiting autologous immune response is beneficial to disease prognosis. Besides, MDSCs were surprisingly proven to have some proimmune functions as well as nerve system regeneration promoting capacities in autoimmune diseases, indicating that MDSC cannot be merely considered as an immunosuppressive cell, and further investigations of its potential functions are necessary. Applying the immunosuppressive property of MDSCs to treat inflammation through in vitro MDSC induction and amplification is another novel field that draws much attention. A number of studies have already achieved artificial MDSC induction by adding cytokines, and the process is accessible theoretically. However, how to systematically induce high‐quality anti‐inflammatory MDSCs at a low cost remains unclear.

MDSCs have a critical role in transforming TME into a protumoral environment, and the main mechanism is to modulate the proliferation and functions of multifarious immune cells. In the current literature, we have summarized the various immunosuppressive functions of MDSCs and the corresponding mechanisms, and we finally established an integrated MDSC interaction network based on previous research results. However, the explorations on MDSC generation, differentiation, and functional mechanisms are still insufficient. Future work is required and is vital to improve clinical antitumor treatment.

Anti‐MDSC effects have recently shown for many targeted antitumor drugs, including some widely used mAbs, small‐molecular inhibitors, and peptides. And we have generalized their functions into five issues: (1) inhibiting MDSC functions, (2) inhibiting MDSC recruitment into TME, (3) restraining myelopoiesis or MDSC development, (4) inducing MDSC differentiation or inhibiting dedifferentiation into MDSCs, and (5) mediating MDSC depletion. Our summary will provide comprehensive references and instructions for clinical drug applications. However, the detailed mechanisms behind the clinical benefits of MDSC‐targeted therapy are poorly understood for most drugs. Further studies should evaluate drug‐MDSC interaction modes, including direct/indirect targeting, or outcomes of secondary tumor‐targeted changes. Since only a small amount of MDSCs exist under steady‐state and normal conditions, MDSC‐targeted therapy can be much less harmful to healthy tissues and cells than traditional chemotherapies, which therefore prevents severe side effects during anticancer treatments.

In the future, after further investigation into MDSCs and their mechanisms in the immune microenvironment, MDSC‐targeted anticancer therapy and MDSC‐based anti‐inflammatory treatment may be finally applied.

## AUTHOR CONTRIBUTIONS

B. S. and R. Y. R. designed the project. R. Y. R., C. Y. X., R. Y. M., and Y. X. W. drafted the manuscript. R. Y. R., C. Y. X., R. Y. M., Y. X. W., and T. Y. Y. finished the search of data for the article. B. S., R. Y. R., and J. Y. Y. revised and finalized the manuscript. All authors read and approved the final manuscript.

## CONFLICT OF INTEREST STATEMENT

The authors declare that they have no competing interests.

## ETHICS APPROVAL AND CONSENT TO PARTICIPATE

Not applicable.

## Data Availability

Not applicable.
